# Electrosynthesis of polymer-grade ethylene via acetylene semihydrogenation over undercoordinated Cu nanodots

**DOI:** 10.1038/s41467-023-37821-1

**Published:** 2023-04-14

**Authors:** Weiqing Xue, Xinyan Liu, Chunxiao Liu, Xinyan Zhang, Jiawei Li, Zhengwu Yang, Peixin Cui, Hong-Jie Peng, Qiu Jiang, Hongliang Li, Pengping Xu, Tingting Zheng, Chuan Xia, Jie Zeng

**Affiliations:** 1 Hefei National Research Center for Physical Sciences at the Microscale, Key Laboratory of Strongly-Coupled Quantum Matter Physics of Chinese Academy of Sciences, Key Laboratory of Surface and Interface Chemistry and Energy Catalysis of Anhui Higher Education Institutes, Department of Chemical Physics, University of Science and Technology of China, 230026 Hefei, Anhui P. R. China; 2 School of Materials and Energy, University of Electronic Science and Technology of China, 611731 Chengdu, P. R. China; 3 Institute of Fundamental and Frontier Sciences, University of Electronic Science and Technology of China, 611731 Chengdu, P. R. China; 4 Key Laboratory of Soil Environment and Pollution Remediation, Institute of Soil Science, Chinese Academy of Sciences, 210008 Nanjing, P. R. China; 5 Yangtze Delta Region Institute (Huzhou), University of Electronic Science and Technology of China, 313001 Huzhou, Zhejiang P. R. China; 6 Institute of Advanced Technology, University of Science and Technology of China, 230031 Hefei, Anhui P. R. China; 7 Research Center for Carbon-Neutral Environmental & Energy Technology, University of Electronic Science and Technology of China, 611731 Chengdu, P. R. China; 8 School of Chemistry & Chemical Engineering, Anhui University of Technology, 243002 Ma’anshan, Anhui P. R. China

**Keywords:** Electrocatalysis, Catalytic mechanisms, Electrocatalysis, Chemical synthesis

## Abstract

The removal of acetylene impurities remains important yet challenging to the ethylene downstream industry. Current thermocatalytic semihydrogenation processes require high temperature and excess hydrogen to guarantee complete acetylene conversion. For this reason, renewable electricity-based electrocatalytic semihydrogenation of acetylene over Cu-based catalysts is an attractive route compared to the energy-intensive thermocatalytic processes. However, active Cu electrocatalysts still face competition from side reactions and often require high overpotentials. Here, we present an undercoordinated Cu nanodots catalyst with an onset potential of −0.15 V versus reversible hydrogen electrode that can exclusively convert C_2_H_2_ to C_2_H_4_ with a maximum Faradaic efficiency of ~95.9% and high intrinsic activity in excess of −450 mA cm^−2^ under pure C_2_H_2_ flow. Subsequently, we successfully demonstrate simulated crude ethylene purification, continuously producing polymer-grade C_2_H_4_ with <1 ppm C_2_H_2_ for 130 h at a space velocity of 1.35 × 10^5 ^ml g_cat_^−1^ h^−1^. Theoretical calculations and in situ spectroscopies reveal a lower energy barrier for acetylene semihydrogenation over undercoordinated Cu sites than nondefective Cu surface, resulting in the excellent C_2_H_2_-to-C_2_H_4_ catalytic activity of Cu nanodots.

## Abstract (web summary)

Cu-based catalysts for the conversion of C_2_H_2_ to C_2_H_4_ are plagued by side reactions. Here, Cu nanodots for C_2_H_2_ semihydrogenation are reported to reach current densities >400 mA cm^−2^ and selectively produce polymer-grade ethylene over 130 h.

## Introduction

Ethylene (C_2_H_4_) is the primary building block in the production of commercially useful commodity chemicals like plastics, antifreeze, fibers, organic solvents, etc.^1–3^. Polyethylene plastic, produced by the *Ziegler-Natta* polymerization process, is one of the most important downstream products of C_2_H_4_. It is worth noting that such a process requires polymer-grade C_2_H_4_ as the feedstock because even 0.5% acetylene (C_2_H_2_) impurities in raw ethylene streams can irreversibly poison the *Ziegler-Natta* catalysts, resulting in much nerfed catalytic activity^4^. Accordingly, to perform this reaction economically at scale in the long term, the C_2_H_2_ impurities in the C_2_H_4_ stream must be lower than 5 parts per million (ppm) to satisfy the polymer-grade C_2_H_4_ requirement^5^. Despite the widespread use of polymer-grade C_2_H_4_ as industrial raw materials, its commercial synthesis still faces inefficient and energy-intensive purification steps.

Typically, solvent absorption and thermally catalyzed semihydrogenation of C_2_H_2_ are the two main strategies to remove C_2_H_2_ impurities from ethylene streams. Solvent absorption is the purification approach in the early years, in which C_2_H_2_ is extracted by solvents such as *N,N*-dimethylformamide, *N*-methylpyrrolidinone, or ethyl acetate. Unfortunately, such a method is not environmentally sustainable due to high-cost organic solvent consumption and C_2_H_4_ loss^6,7^. Even though emerging porous sorbents, especially metal-organic frameworks, exhibit the potential for C_2_H_2_/C_2_H_4_ separation^8–12^, it also suffers from several drawbacks, such as poor stability and the trade-off between adsorption capacity and selectivity^1^. Currently, thermocatalytic semihydrogenation of C_2_H_2_ over Pd-based catalysts is widely used for the industrial-scale synthesis of polymer-grade C_2_H_4_^13–20^, but it requires relatively high operating temperatures (100–250 °C). In addition, excess hydrogen (H_2_) is needed to guarantee this conversion process, thereby bringing safety problems and leading to over-hydrogenation. Further, an energy-intensive downstream separation is often required to obtain clean C_2_H_4_. Therefore, a cost-effective and sustainable strategy is desirable to purify the crude C_2_H_4_ into polymer-grade feedstocks.

Electrocatalytic acetylene semihydrogenation (EASH) is an appealing alternative approach (C_2_H_2_ + 2H_2_O + 2e^−^ → C_2_H_4_ + 2OH^−^) powered by renewable electricity under ambient conditions. EASH can be carried out in aqueous media, and water (H_2_O) serves as the proton source rather than excess H_2_, which is in line with the concept of safety and sustainability. Nonetheless, due to the poor solubility of acetylene and lack of efficient catalysts, EASH has been stagnant since its appearance in the 1970s^21–23^. Recent works have addressed the solubility issue using gas diffusion layer (GDL) electrodes, where the mass transfer limitations can be broken by constructing a triple-phase boundary^24–27^. However, it is still essential to deal with the competing hydrogen evolution reaction (HER), over-hydrogenation, and C–C coupling. Copper (Cu) has been recognized as one of the predominant catalysts for activating acetylene while selectively producing C_2_H_4_. Despite the vigorous efforts in engineering Cu catalysts towards efficient EASH, these reported Cu catalysts required high overpotentials^24–26^, translating to lower energy efficiency, and failed to completely remove the C_2_H_2_ impurities (<1 ppm) from the crude ethylene flow.

Here, we designed undercoordinated Cu nanodots (Cu NDs) as highly efficient EASH catalyst, which was derived from in situ reduction supplied with C_2_H_2_ gas. Under pure acetylene flow in a three-electrode flow cell, the Cu NDs exhibited a relatively positive onset potential of −0.15 V vs. RHE and achieved a maximum ethylene Faradaic efficiency (FE_C2H4_) of 95.9% at −0.69 V vs. RHE. The FE_C2H4_ remained above 90.4% with a C_2_H_4_ partial current density of −452 mA cm^−2^, superior to the reported catalysts^3,24–26^. Theoretical calculations and in situ spectroscopies revealed that the impressive catalytic activity of Cu NDs could be ascribed to its lower rate-determining-step energy barrier for acetylene semihydrogenation on undercoordinated Cu sites. We further showcased the continuous production of polymer-grade C_2_H_4_ from simulated crude gas that contains 0.5% acetylene. Under a flow rate of 50 standard cubic centimeter per minute (sccm), corresponding to a space velocity of 1.35 × 10^5^ ml g_cat_^−1^ h^−1^, our homemade reactor with 25 cm^2^ electrode area could continuously generate ultrapure C_2_H_4_ (C_2_H_2_ < 1 ppm) for at least 130 h.

## Results

### Structural characterization of the Cu NDs electrocatalyst

**Fig. 1:**
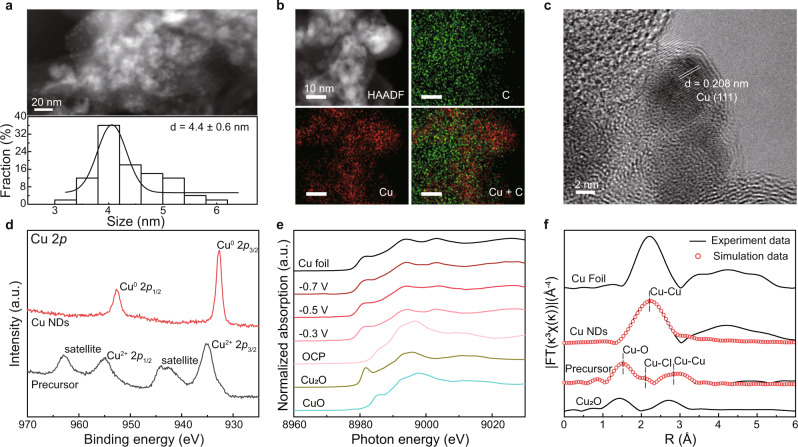
Structural characterization of the Cu NDs catalyst. **a** HAADF-STEM image of Cu NDs (up) and the corresponding size distribution histograms (down). **b** STEM-EDX mapping of the Cu NDs catalyst. **c** HRTEM image of the Cu NDs catalyst. **d** Cu 2*p* XPS spectra of the precursor and Cu NDs catalyst. **e** Normalized in situ XANES spectra of the Cu NDs catalyst under EASH conditions at different working potentials vs. RHE, along with the spectra of Cu foil, Cu_2_O, and CuO as references. OCP, open-circuit potential. **f** Cu *K*-edge phase-uncorrected EXAFS spectra and the corresponding simulation curves in R space of the precursor and Cu NDs catalyst, along with the spectra of Cu foil and Cu_2_O as references.

We first synthesized the copper precursor using a facile hydrolysis method (see the “Methods” section), which yielded carbon-supported Cu_2_Cl(OH)_3_ nanoparticles (Supplementary Figs. 1–5). To form the Cu NDs catalyst, we sprayed the precursor onto the GDL, which was subjected to the in situ reduction under a constant current density of −100 mA cm^−2^ for 30 min in a standard three-electrode flow cell under pure C_2_H_2_ flow. The as-reduced samples were then peeled off from the GDL for further structural characterization. Transmission electron microscopy (TEM) and high-angle annular dark-field scanning transmission electron microscopy (HAADF-STEM) images, in conjunction with energy dispersive X-ray (EDX) elemental mapping, clearly showed the uniform dispersion of Cu nanodots on activated carbon particles, with an average size of 4.4 ± 0.6 nm (Fig. 1a, b). As shown in the high-resolution TEM (HRTEM) image (Fig. 1c), the lattice fringe spacing of 0.208 nm marked on the nanodots can be indexed to the (111) plane of Cu^28^. The X-ray diffraction (XRD) and selected area electron diffraction (SAED) (Supplementary Figs. 6, 7) further revealed a pure metallic Cu crystal structure for the as-reduced Cu NDs^29^. X-ray photoelectron spectroscopy (XPS) reconfirmed the Cu (0) valence state of the Cu NDs (Fig. 1d and Supplementary Fig. 8)^30^. The mass loading of Cu in the carbon-supported Cu NDs catalyst was determined to be 29.04 wt% by inductively coupled plasma atomic emission spectroscopy (ICP-AES).

In an effort to investigate the electronic structure and coordination environment of Cu NDs under EASH conditions, we then conducted in situ X-ray absorption spectroscopy (XAS) measurements. Figure 1e shows the Cu *K*-edge normalized X-ray absorption near-edge spectroscopy (XANES) profiles of Cu NDs at different working potentials, along with Cu foil, Cu_2_O, and CuO as references. The white line of Cu NDs at different applied potentials resembled that of copper foil, suggesting that Cu in NDs catalyst maintained a metallic feature under EASH condition^31^. The results of XANES deconvolution by linear combination fitting of the spectra of Cu^0^ and Cu^+^ also confirmed the active sites in the EASH were metallic Cu nanodots (Supplementary Fig. 9). As shown in the extended X-ray absorption fine structure (EXAFS) spectra (Fig. 1f and Supplementary Fig. 10), the prominent peaks of Cu NDs under different working potentials locate at approximately 2.21 Å, which was ascribed to the Cu–Cu bonds. In contrast, the spectrum of the precursor showed characteristic fingerprint peaks of Cu–O, Cu–Cl, and Cu–Cu coordination. These results were also corroborated by wavelet transform analysis and the fitting data (Supplementary Fig. 11 and Supplementary Table 1). It is worth noting that the Cu–Cu coordination number of Cu NDs at different applied potentials was ~10, lower than that of 12 for Cu foil (Supplementary Table 1), implicating the unsaturated coordination of Cu sites for Cu NDs. The above results, taken together, suggest that the active phase of our in situ formed Cu NDs under EASH conditions is metallic Cu featuring undercoordinated sites, which could be represented by stepped Cu (211)^32^.

### Acetylene electroreduction under pure acetylene flow

**Fig. 2:**
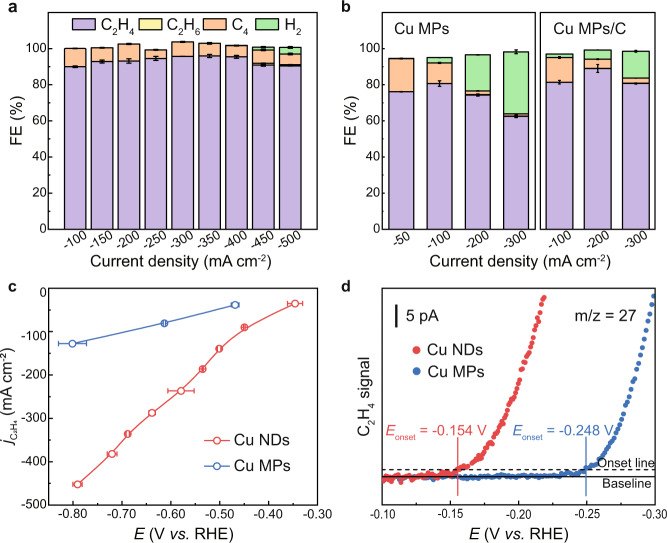
Electrocatalytic acetylene semihydrogenation performance over Cu-based electrocatalysts under pure acetylene flow. **a**, **b** FEs of EASH products at different current densities of **a** Cu NDs and **b** Cu MPs, Cu MPs/C. **c** Variation in the ethylene partial current density against applied potential over Cu NDs and Cu MPs. **d** In situ DEMS measurement of C_2_H_4_ production during EASH over Cu NDs and Cu MPs. All tests were conducted using a three-electrode flow cell in 1 M KOH solution at room temperature under pure acetylene flow. The error bars correspond to the standard deviation of at least three independent measurements.

The acetylene-to-ethylene performance of the Cu NDs was first evaluated under pure acetylene flow at the flow rate of 30 sccm in a typical three-electrode flow cell, with 1 M KOH as the electrolyte (see the “Methods” section, Supplementary Figs. 12, and 13). As revealed by linear sweep voltammetry (LSV) in Supplementary Fig. 14, the Cu NDs afforded a notably higher current density under pure acetylene than that under Ar, indicating the participation of C_2_H_2_ in the reaction. Steady-state chronopotentiometry of acetylene electrolysis was recorded under different current densities from −100 to −500 mA cm^−2^. The gas and liquid products at different current densities were analyzed and quantified by gas chromatography (GC) and nuclear magnetic resonance (NMR), respectively. We found that C_2_H_4_ was the main product over Cu NDs during EASH, along with a small amount of C_4_ olefin and negligible H_2_ (Fig. 2a and Supplementary Table 2). No other liquid products were detected according to the ^1^H-NMR spectrum (Supplementary Fig. 15). For Cu NDs, a high plateau of C_2_H_4_ Faradaic efficiency over 90% was retained under a broad potential range from −0.45 to −0.79 V vs. RHE. The maximum C_2_H_4_ FE of ~95.9% was achieved at a current density of −350 mA cm^−2^ under −0.69 V vs. RHE. Notably, the FE of the competitive HER was suppressed to below 0.1% until the current density ascended to −400 mA cm^−2^. In addition, Cu NDs exhibited excellent stability under EASH conditions (Supplementary Fig. 16) and the effect of copper mass loading in Cu NDs on C_2_H_2_-to-C_2_H_4_ performance was also evaluated in Supplementary Fig. 17.

For comparison, the EASH catalytic activities of Cu microparticles (Cu MPs, Supplementary Figs. 18, 19) and Cu MPs loaded on activated carbon (Cu MPs/C) were investigated under the same test conditions as those of Cu NDs (Fig. 2b and Supplementary Fig. 20). In the case of Cu MPs, the maximum C_2_H_4_ FE was 85.0% at the current density of −150 mA cm^−2^, which decreased rapidly as the current density increased due to HER competition, with the H_2_ FE in excess of 34% at −300 mA cm^−2^. The same trend was observed for Cu MPs/C, suggesting activated carbon particles did not contribute to the improvement of EASH performance. Figure 2c depicts the ethylene partial current density (*j*_C2H4_) at different potentials of Cu NDs and Cu MPs. The curves showed that the catalytic activity of Cu NDs was significantly higher than that of Cu MPs under the same working potential, as exemplified by the higher *j*_C2H4_ of Cu NDs. Notably, an impressive *j*_C2H4_ of Cu NDs was achieved as high as −452 mA cm^−2^ at −0.79 V vs. RHE, while the C_2_H_4_ FE remained above 90%. We also conducted in situ differential electrochemical mass spectrometry (DEMS) over Cu NDs and Cu MPs under pure acetylene, where the onset potential of Cu NDs was about −0.15 V vs. RHE, which was ~94 mV more positive than that of Cu MPs (Fig. 2d). This comparison presented that the undercoordinated Cu NDs catalyst exhibited higher intrinsic activity for EASH than its bulk counterparts. The same conclusion could be drawn from LSV curves, which also suggested the negligible contribution of GDL to EASH activity (Supplementary Fig. 21). As shown in Supplementary Fig. 22 and Supplementary Table 3, our Cu NDs catalyst shows superiorities with respect to high *j*_C2H4_ and high FE_C2H4_ compared with previously reported catalysts^3,21,23–26,33,34^.

### Study of the reaction mechanism

**Fig. 3:**
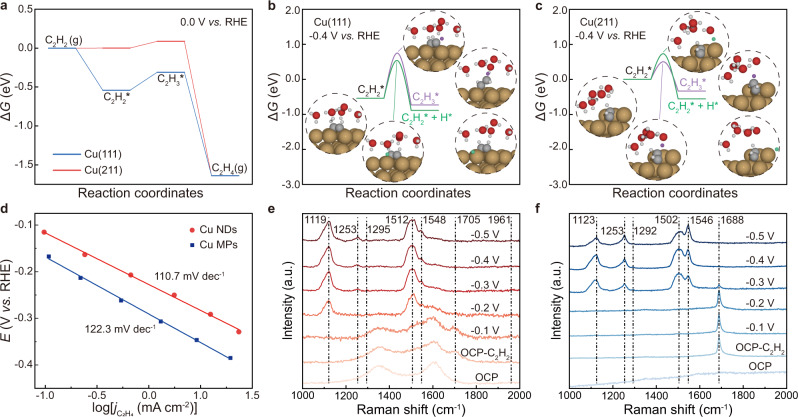
DFT theoretical calculations and in situ electrochemical Raman study. **a** Free energy diagram of reducing C_2_H_2_ on Cu (111) and Cu (211) surfaces. **b**, **c** Comparison between the barriers of forming C_2_H_3_* and H* on **b** Cu (111) and **c** Cu (211) surfaces. All energies were referenced to C_2_H_2_ and a computational hydrogen electrode (CHE). **d** Tafel plots for EASH over Cu NDs and Cu MPs. **e**, **f** In situ electrochemical Raman spectra of **e** Cu NDs and **f** Cu MPs in a 1 M KOH solution at different working potentials vs. RHE without IR compensation. OCP open-circuit potential.

Density functional theory (DFT) simulations were first carried out to elucidate the reaction mechanism of C_2_H_2_ electroreduction on Cu. Herein, two types of surfaces were investigated: the Cu (111) surface representing the flat, nondefective surface and the stepped Cu (211) surface featuring undercoordinated sites created by either inherent crystal orientations or local defects^32^. The reaction free energy of forming C_2_H_4_ from C_2_H_2_ was first compared in Fig. 3a under 0 V vs. RHE, equivalent to a computational hydrogen electrode (CHE) by definition. For both surfaces, the potential limiting step (PLS) is the reduction of adsorbed C_2_H_2_* to C_2_H_3_* because of the relatively strong adsorption of C_2_H_2_* on both surfaces. In particular, Cu (111) exhibited significantly stronger stabilization to C_2_H_2_* than Cu (211) and more negative potential is required for driving the above-identified PLS. In other words, undercoordinated Cu sites were able to catalyze C_2_H_2_ electroreduction at lower overpotentials than terrace Cu sites, which was consistent with the DEMS results (Fig. 2d). To further understand the C_2_H_4_ selectivity against HER, the kinetic barriers of proton transfer to C_2_H_2_* and forming C_2_H_3_* were compared with the barriers of transferring a proton to the surface and forming H* (Fig. 3b, c). The as-formed H* then produced H_2_ through either Heyrovsky or Tafel pathways. It was clear that under −0.4 V vs. RHE, a potential where a reasonably large current density was observed experimentally, the barrier of forming C_2_H_3_* on the Cu (211) surface (0.49 eV) was considerably lower than that on the Cu (111) surface (1.29 eV). This barrier was also smaller than the barrier of forming H* on the Cu (211) surface (0.72 eV). In contrast, an inverse trend was observed for the Cu (111) surface. The same conclusion could also be drawn from the Tafel plots, where the EASH over Cu NDs and Cu MPs have the same rate-determining step (110.7 mV dec^−1^ for Cu NDs and 122.3 mV dec^−1^ for Cu MPs), and the lower Tafel slope on Cu NDs indicates that the hydrogenation of adsorbed C_2_H_2_* to C_2_H_3_* has faster reaction kinetics (Fig. 3d). Thus, the undercoordinated sites of Cu are identified as the active sites for selective C_2_H_2_ electroreduction against the HER. As disclosed by the XAFS fitting results (Supplementary Table 1), Cu NDs feature abundant undercoordinated sites, thus rendering high activity for selective C_2_H_2_ electroreduction. To rule out the effect of surface area and obtain a clearer idea of the intrinsic activity, we conducted Pb underpotential deposition (UPD) to obtain the electrochemically active surface area (ECSA) (Supplementary Fig. 23)^35^. The ECSA-normalized current densities of Cu NDs were still higher than those of Cu MPs under the same working potential, indicating the superior intrinsic catalytic activity of Cu NDs over Cu MPs (Supplementary Fig. 24). Moreover, Cu NDs intrinsically favored selective C_2_H_4_ production, while Cu MPs was more preferable to HER (Supplementary Fig. 25).

We further conducted in situ electrochemical Raman spectroscopy to monitor the EASH processes on these Cu catalysts (Fig. 3e, f). For Cu NDs, at open-circuit potential (OCP), the peaks at approximately 1350 and 1610 cm^−1^ belonged to the D-Peak and G-Peak of carbon^36^, respectively. Once C_2_H_2_ was introduced, a fingerprint peak corresponding to absorbed C_2_H_2_* appeared at 1705 cm^−1^, which exhibited a red-shift relative to C_2_H_2_ (1961 cm^−1^)^37^, associated with the σ-π-C_2_H_2_ adsorbed configuration^38^. When increasing the potentials for Cu NDs, the intensity of the C_2_H_2_* peak decreased gradually, suggesting that C_2_H_2_ was being consumed (Fig. 3e). Upon applying the cathodic potential to −0.2 V vs. RHE, the π-d type adsorbate-surface ethylene signal arose, including 1548 cm^−1^ for the C = C stretching mode and 1295 cm^−1^ for the CH_2_ bending mode^39^. Beyond that, additional peaks were observed at 1119, 1253, and 1512 cm^−1^, which were assigned to the C–C, C–H, and C = C vibrations of polyacetylene^38^, respectively (Fig. 3e). In contrast, the signal of C_2_H_2_* over Cu MPs was observed at 1688 cm^−1^ (Fig. 3f), indicating the same adsorbed configuration but a stronger adsorption strength than Cu NDs, conforming to the DFT simulation results (Fig. 3a). In addition, the ethylene vibration signals over Cu MPs were observed at 1546 and 1292 cm^−1^, which were red-shifted compared with that over Cu NDs, suggesting the stronger adsorption of C_2_H_3_* over Cu MPs. These results indicated that ethylene was more readily desorbed from Cu NDs than Cu MPs and thus Cu NDs had better ethylene selectivity.

### Electrosynthesis of polymer-grade C_2_H_4_

Considering that the concentration of acetylene impurities in industrial crude ethylene is generally between 0.5% and 3%, we, therefore, employed an ethylene-rich gas source (0.5% C_2_H_2_, 20% C_2_H_4_ balanced with Ar) to simulate the crude ethylene. A three-electrode flow cell was first used to evaluate the EASH catalytic activity over different catalysts at low acetylene partial pressure. As shown in Supplementary Fig. 26, at the flow rate of 20 sccm, a peak C_2_H_2_ conversion of 67.2% was afforded over Cu NDs, which was higher than that of 43.4% over Cu MPs. Ethylene was the major product of the EASH reaction for Cu NDs, with the highest C_2_H_4_ FE of 92.0% at −10.0 mA, while HER was suppressed over Cu NDs to a greater extent than over Cu MPs.

**Fig. 4:**
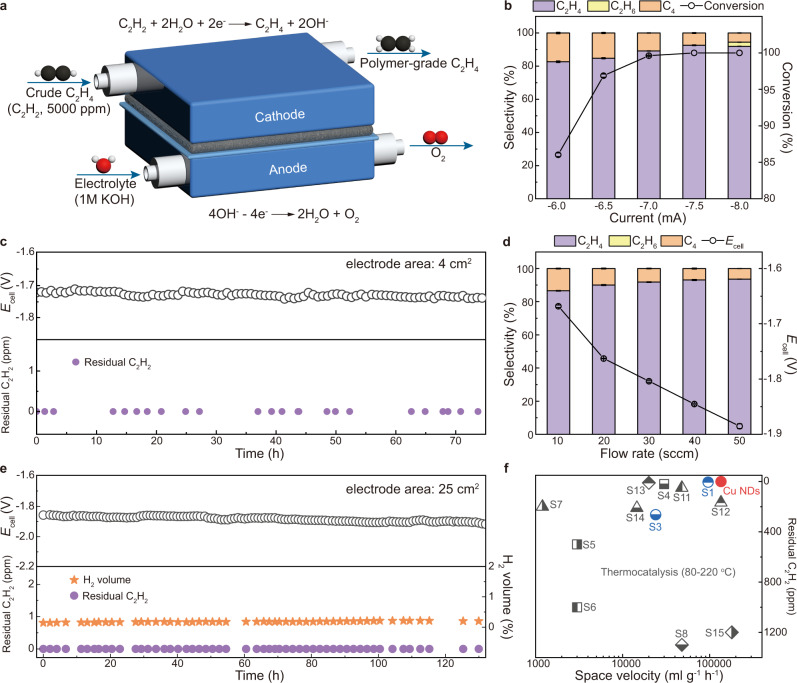
Acetylene removal in ethylene-rich simulated gas (0.5% C_2_H_2_, 20% C_2_H_4_ balanced with Ar). **a** Schematic of the homemade MEA-type two-electrode reactor. Between the cathode and anode were the GDL loaded with catalyst and anion exchange membrane (AEM). **b** Selectivity of EASH products at different currents and corresponding C_2_H_2_ conversion over Cu NDs in the two-electrode reactor (electrode area, 4 cm^2^) at a flow rate of 10 sccm. **c** Long-term operation of EASH at a constant current of −7.50 mA under the reaction conditions of (**b**). **d** Selectivity of EASH products at different flow rates and the corresponding full-cell potential over Cu NDs in the homemade 25 cm^2^ two-electrode reactor. Applied current: 10 sccm, −8.0 mA; 20 sccm, −18.0 mA; 30 sccm, −28.0 mA; 40 sccm, −39.0 mA; 50 sccm, −50.0 mA. **e** Durability test for 130 h conducted in the homemade 25 cm^2^ two-electrode reactor at the current of −50 mA with the flow rate of 50 sccm. **f** Comparison of the acetylene semihydrogenation performance over Cu NDs with previously reported state-of-the-art catalysts. The balls represent the electrocatalytic processes while the others were the thermocatalysis. All comparison data are from the references summarized in Supplementary Table 5.

Taking advantage of low resistance, low energy consumption, and compact configuration, membrane electrode assembly (MEA) reactors are promising for performing practical gas-phase reactions. As such, we designed an MEA-type two-electrode reactor (Fig. 4a) for the continuous generation of polymer-grade C_2_H_4_. Figure 4b shows the EASH products distribution over Cu NDs at different currents, along with the corresponding C_2_H_2_ conversion rate, in a two-electrode MEA reactor (electrode area, 4 cm^2^) at a flow rate of 10 sccm. When a current of −7.50 mA was applied (theoretical limiting conversion current is −6.98 mA, see the “Methods” section), acetylene could be completely reduced (<1 ppm, Supplementary Fig. 27) with 92.6% C_2_H_4_ selectivity, while HER was entirely stifled (Supplementary Fig. 28). Remarkably, our two-electrode MEA reactor can steadily operate for as long as 70 h at −7.50 mA without noticeable performance decay, continuously providing ultrapure C_2_H_4_ with negligible gas impurities (<1 ppm for acetylene, ethane, and hydrogen, Fig. 4c). In addition, Cu NDs maintained its morphology, phase, and valence state after the stability test, indicating its excellent material stability (Supplementary Fig. 29).

To validate the scalability of our MEA device and achieve a higher ethylene purification efficiency, we extended the geometric electrode area from 4 cm^2^ used for performance evaluation to 25 cm^2^ in one-unit modular cell (Supplementary Fig. 30). As depicted in Fig. 4d, the enlarged MEA reactor could completely convert C_2_H_2_ impurities to C_2_H_4_ at different flow rates from 10 to 50 sccm to meet various production requirements. In an aim to remove the acetylene as thoroughly as possible, we applied a current slightly higher than the corresponding theoretical limiting conversion current at different flow rates. This showed that with the increase in flow rate, the selectivity of ethylene took on an upward trend, where the ethylene selectivity remained above 90% at a flow rate >20 sccm and reached 93.6% at 50 sccm, corresponding to a space velocity of 1.35 × 10^5^ ml g_cat_^−1^ h^−1^. In addition, because of the relatively positive onset potential, Cu NDs showed a cell voltage of −1.89 V at 50 sccm, leading to improved reactor energy efficiency. Higher flow rates also resulted in a slight increase of hydrogen volume in the ethylene flow, presumably due to the shortened gas residence time and the applied higher current, which both encouraged the HER (Supplementary Table 4). Notably, our enlarged two-electrode reactor can continuously operate for 130 h at −50.0 mA with negligible performance decay (residual C_2_H_2_ < 1 ppm, H_2_ volume ≈ 0.18%, cell voltage maintained at approximately −1.89 V), producing ultrapure C_2_H_4_ along with 1800 ppm H_2_ and 370 ppm C_4_ species (Fig. 4e), which can be easily separated using the current cryogenic liquefaction apparatus (Supplementary Fig. 31). As shown in Fig. 4f, Supplementary Fig. 32, Supplementary Tables 5, and  6, Cu NDs exhibits distinct advantages over reported state-of-the-art catalysts in C_2_H_2_ removal. These results cast light on the potential of electrocatalytic acetylene semihydrogenation over Cu NDs for the production of a polymer-grade ethylene feed. Moreover, the modularity and ease of scale-up of the MEA reactor used in our work provide an unprecedented opportunity to accelerate the industrialization of this approach (Supplementary Note 1).

## Discussion

By virtue of the experimental and theoretical results, we demonstrated that the undercoordinated Cu NDs catalyst exhibited remarkable catalytic activity for EASH with a relatively positive onset potential of −0.15 V vs. RHE and a high FE_C2H4_ of 90.4% under the high C_2_H_4_ partial current density of −452 mA cm^−1^. Moreover, we showcased the electrocatalytic purification of crude ethylene via a homemade two-electrode MEA reactor at a space velocity of 1.35 × 10^5^ ml g_cat_^−1^ h^−1^ and resulted in negligible acetylene impurities (<1 ppm). Our work implicates a great potential for replacing the current energy-intensive thermal catalysis process and provides a sustainable avenue for highly efficient electricity-powered acetylene-to-ethylene conversion.

## Methods

### Chemicals

All chemicals were used without further purification. Copper (II) chloride (CuCl_2_), copper sulfate pentahydrate (CuSO_4_·5H_2_O), ascorbic acid (AA), sodium hydroxide (NaOH), potassium hydroxide (KOH), sodium chloride (NaCl), ethanol (>99.7%), isopropanol (>99.7%), diethanolamine (DEA > 99%), ethylene glycol (EG > 99%) were purchased from Macklin. Polyvinylpyrrolidone (PVP, K29-K32) and Nafion (5 wt%) were purchased from Sigma-Aldrich. Activated carbon (Vulcan XC-72) was obtained from SCI Materials Hub. Deionized (DI) water was used throughout the experiments.

### Preparation of Cu NDs

For the synthesis of carbon-supported Cu_2_Cl(OH)_3_ precursor, 400 mg of activated carbon (XC-72) was dispersed in 80 ml of ethanol in a 250 ml flask with sonication for 30 min. After that, 1 g of CuCl_2_ was added into the flask and stirred for another 30 min to get a uniform ink. Diethanolamine (DEA) solution (1 g of DEA dissolved in 5 ml of ethanol) was then slowly dropped into the flask for 10 min and refluxed at 100 °C for 1 h, under stirring. After cooling to room temperature, 100 ml of DI water was added into the flask and sonicated for 30 min to completely hydrolyze the copper complex. The precursor was collected by centrifugation at 15,800 × *g* for 10 min and washed three times with DI water and two times with ethanol, respectively, and then dried at 70 °C for 24 h. Cu NDs catalyst was then in situ formed by reducing the precursors at a constant current density of −100 mA cm^−2^ for 30 min in a standard three-electrode flow cell system supplied with C_2_H_2_ gas at a flow rate of 30 sccm, using 1 M KOH as electrolyte. The mass loading of copper in the Cu NDs can be controlled easily by adjusting the amount of CuCl_2_ in the feed.

### Preparation of Cu MPs

To synthesize the Cu MPs, 125 mg of CuSO_4_·5H_2_O and 200 mg of PVP were dissolved in a 50 ml flask containing 20 mL of EG. Then added 0.2 mL of NaCl EG solution (1 M) into the flask and heated to 100 °C. Next, another mixture of 1 mL of NaOH EG solution (1 M) and 2.5 mL of AA EG solution (0.5 M) was slowly injected into the flask. Then the mixture was heated for 40 min at 100 °C. After that, Cu MPs were cleaned and collected using a mixture of ethanol and water by centrifugation.

### Preparation of working electrodes

30 mg of the catalyst precursor powder was dispersed in 6 ml of isopropanol with sonication for 1 h. Then, 40 μl of Nafion solution (5 wt%) was added into the solution for another 30 min. After that, the precursor ink was air-brushed onto a piece of 4 × 4 cm^2^ GDL (YLS-30T) to obtain the working electrode. The mass loading of catalysts precursors on GDL was controlled to be 1 mg cm^−2^, after the effect of mass loading per geometric area on acetylene-to-ethylene performance was evaluated by controlling the mass loading of precursors on GDL at 0.49, 0.89, and 2.02 mg cm^−2^, respectively (Supplementary Figs. 12 and 13).

### Electrochemical measurements

All electrochemical measurements were conducted at room temperature. CHI (1140c) and Bio-Logic (VSP-3e) electrochemical workstations were employed for the electrochemical measurements. The Ag/AgCl (saturated KCl) was adopted as the reference electrode, and the counter electrode was Ni foam for the oxygen evolution reaction. All potentials were converted to the RHE reference scale using the relation below and the solution resistance was compensated with an 80% compensation coefficient unless otherwise mentioned.

1 $${E}_{{{{{{\rm{RHE}}}}}}}={E}_{{{{{{\rm{Ag}}}}}}/{{{{{\rm{AgCl}}}}}}}+0.198+0.059\times {{{{{\rm{pH}}}}}}$$

For a three-electrode flow cell system under pure C_2_H_2_, a customized reactor with an electrode area of 0.75 cm^2^ was designed. The cathode gas chamber was supplied with pure C_2_H_2_ at a flow rate of 30 sccm, when 1 M KOH was pumped around both the cathode and anode side at a flow rate of 4 ml min^−1^. A Nafion 115 membrane (Fuel Cell Store) was sandwiched between an anolyte and electrolyte to separate the chambers.

For three-electrode flow cell systems under ethylene-rich simulated gas (0.5% C_2_H_2_, 20% C_2_H_4_ balanced with Ar), the experimental conditions were consistent with the above except that the flow rate was 20 sccm.

For two-electrode reactors, two types of electrode area (2 × 2 and 5 × 5 cm^2^) were used to purify ethylene. Feed gas (0.5% C_2_H_2_, 20% C_2_H_4_ balanced with Ar) was delivered to the GDL from the back side of the cathode at the different flow rate, while 1 M KOH aqueous solution was circulated around the anode side at a flow rate of 10 ml min^−1^. An anion-exchange membrane (AEM, Dioxide Materials and Membranes International) was used for ion exchange.

In situ DEMS was conducted using a homemade flow cell and the mass spectrometer was equipped with a capillary injection port. The scan rate of LSV measurement was set to 5 mV s^−1^. The signal of mass-to-charge ratio of 27 and 26 was ascribed to C_2_H_4_ and C_2_H_2_, respectively. The onset line was determined according to the positions where the signal-to-noise ratio reached 5.

In situ Raman analyses were performed using the Renishaw inVia Raman analyzer equipped with 532 nm laser, combined with the custom flow cell. While catalysts loaded GDL served as the working electrodes, Pt wire and Ag/AgCl (saturated KCl) were used as counter and reference electrodes, respectively. During experiments, the laser was focused on the surface of the sample with a laser intensity of 5 mW.

Pd UPD was conducted in an Ar-saturated solution of 100 mM HClO_4_ + 1 mM Pb(ClO_4_)_2_, using Pt wire as the anode electrode. The cathode was held at −0.12 V vs. RHE for 1 min and then LSV was taken from −0.12 to 0.10 V vs. RHE at a scan rate of 5 mV s^−1^. The background was recorded in 100 mM HClO_4_ without Pb(ClO_4_)_2_. The ECSA was calculated from the total charge of Pb monolayer stripping from Cu surface with a conversion factor of 310 μC cm^−2^.

### Data analysis

The gas products (H_2_, C_2_H_2_, C_2_H_4_, C_2_H_6_, C_4_) were analyzed using a gas chromatograph (GC, Agilent 8990) coupled with a thermal conductivity detector and flame ionization detector. The calibration curve across the zero point confirmed that the detection concentration limit of GC was as low as 1 ppm (Supplementary Fig. 27). The FE of the gas products was calculated through the concentration (*x*) detected by GC according to the equation:

2 $${{{{{\rm{FE}}}}}}(\%)=\frac{nF{xv}}{{V}_{{\rm {m}}}I}\times \frac{1}{60}\times 100\%$$

where *n* is the electron transfer number, *F* is Faraday constant (96,485 C mol^−1^), *x* is the mole fraction of the product, *v* is the flow rate of gas (sccm), *V*_m_ is the molar volume (24.5 L mol^−1^), and *I* is the applied current (A).

For simulated crude ethylene, because of the abundant ethylene in the feed gas, the FE of C_2_H_4_ was calculated as follows:

3 $${{{{{{\rm{FE}}}}}}}_{{{{{{\rm{ethylene}}}}}}}(\%)=({1-{{{{{\rm{FE}}}}}}}_{{{{{{\rm{other}}}}}}{{{{{\rm{products}}}}}}})\times 100\%$$

where FE_other products_ represent the total FE of other products exclude C_2_H_4_, including H_2_, C_2_H_6_, and C_4_ olefin.

The theoretical limiting conversion current (*I*_limit_) for completely reduce C_2_H_2_ to C_2_H_4_ was calculated as follows:

4 $${I}_{{{{{{\rm{limit}}}}}}}=\frac{nF{vx}}{{V}_{\rm {{m}}}\times 60}$$

where *n* is the electron transfer number (2 for C_2_H_2_ reducing to C_2_H_4_), *F* is Faraday constant (96,485 C mol^−1^), *v* is the flow rate of gas (sccm), *x* is the mole fraction of the C_2_H_2_ and *V*_m_ is the molar volume (24.5 L mol^−1^). For example, in our C_2_H_2_ impurities removal experiments using a two-electrode MEA-type reactor (electrode surface area, 4 cm^2^), *x* = 0.5% and *v* = 10.64 sccm (10 sccm in Ar mode), thus *I*_limit_ = 6.98 mA.

The acetylene conversion and selectivity for ethylene were calculated as follows:

5 $${{{{{\rm{Conversion}}}}}}(\%)=\frac{c-c^{\prime} }{c}\times 100\%$$

6 $${{{{{\rm{Selectivity}}}}}}(\%)=\frac{c-c^{\prime} -[{C}_{2}{H}_{6}]-[{C}_{4}]}{c-c^{\prime} }\times 100\%$$

where *c* represents the acetylene concentration in the feed gas and *c’*, [C_2_H_6_], [C_4_] are the concentration of acetylene, ethane, and C_4_ olefins in the product gas.

The liquid products were quantified by collecting and analyzing the electrolyte using the 400 MHz NMR spectrometer. Typically, 600 μl electrolyte was mixed with 100 μl D_2_O (Sigma Aldrich, 99.9 at.% D) and 0.05 μl dimethylsulfoxide (Sigma Aldrich, 99.9%) as internal standard.

### Characterizations

Scanning electron microscope (SEM) images were taken on Gemini SEM 300 (ZEISS, Germany) at an accelerating voltage of 5 kV. X-ray diffraction (XRD) patterns were recorded using the Shimadzu X-ray Diffractometer (XRD-6100, Japan) with Cu-K_α_ radiation (*λ* = 1.54178 Å). X-ray photoelectron spectroscopy (XPS) measurements were performed on a Kratos-Axis Supra XPS spectrometer with an exciting source of Al K_α_ = 1486.6 eV. The binding energies obtained in the XPS spectral analysis were corrected by referencing C 1*s* to 284.3 eV. Transmission electron microscope (TEM), high-resolution transmission electron microscope (HRTEM), high-angle annular dark-field scanning transmission electron microscope (HAADF-STEM) images, and energy dispersive X-ray (EDX) elemental mapping were carried out on Tecnai G^2^ F20 S-TWIN using Mo-based TEM grids. Fourier transform infrared spectroscopy (FTIR) spectra were obtained at room temperature on a Thermo Fisher Nicolet iS50 ATR spectrometer equipped with an MCT detector. The X-ray absorption spectra (XAS) of Cu *K*-edges were conducted at BL11B beamline of Shanghai Synchrotron Radiation Facility (SSRF) under “top-up” mode with a constant current of 200 mA, recorded under fluorescence mode with a Lytle detector.

### Computational details

The structural optimizations were performed with density functional theory, with a periodic plane-wave implementation using Vienna ab initio Simulation Package (VASP) code^40,41^. The exchange-correlation energy was modelled by using Perdew–Burke–Ernzerhof (PBE) functional^42^ within the generalized gradient approximation (GGA). The projector augmented wave (PAW) pseudo-potentials^43^ were used to describe ionic cores. An energy cutoff of 500 eV was adopted. A first-order Methfessel–Paxton smearing of 0.1 eV was applied to the orbital occupation during the geometry optimization and for the energy computations.

The adsorption energies were evaluated using three-layer 3 × 3 supercells with the bottom two layers constrained, and [4 × 4 × 1] Monkhorst–Pack *k*-point grids were used^44^ with a convergence threshold of 10^–5^ eV for the iteration in the self-consistent field (SCF). All structures were optimized until force components were <0.02 eV/Å. The vibrational frequencies of free molecules and adsorbates were calculated by using the phonon modules in the VASP 5.3 code. A standard thermodynamic correction was applied to determine the free energy corrections, including the correction of the effect from zero-point energy, pressure, inner energy, and entropy.

The transition states were determined using the method of climbing image nudged elastic band (CI-NEB)^45^, with a convergence threshold of 0.05 eV/Å. All structures contained a three-layer metal slab with atoms in the top layer relaxed and the rest fixed, along with an ice-like water structure^46^ for the (111) facets and hydrogen-bonded water layers for the (211) facet determined through minima hopping^47^.

The potential-dependent electrochemical kinetic barriers were obtained through the charge-extrapolation scheme^48,49^. All transition states were referenced to the initial state of aqueous protons and electrons in bulk solution, as determined using the computational hydrogen electrode^50^.

## Supplementary information

Supplementary Information

Peer Review File

## Data Availability

All the data that support the findings of this study are available from the corresponding authors upon reasonable request. Source data are provided with this paper.

## Source data

Source Data
