# Supplementary material for: Electrosynthesis of polymer-grade ethylene via acetylene semihydrogenation over undercoordinated Cu nanodots
Source: Nat Commun. 2023 Apr 14;14:2137. doi: 10.1038/s41467-023-37821-1 (PMC10104804; doi:10.1038/s41467-023-37821-1)
Supplement: Supplementary file 1 — Supplementary Information [file 41467_2023_37821_MOESM1_ESM.pdf]

Supplementary Information for

Electrosynthesis of polymer-grade ethylene via acetylene semihydrogenation over undercoordinated Cu nanodots

Weiqing Xue[1,2]†, Xinyan Liu[3]†, Chunxiao Liu[2], Xinyan Zhang[1,2], Jiawei Li[1,2], Zhengwu Yang[1], Peixin Cui[4], Hong-Jie Peng[3,5], Qiu Jiang[2], Hongliang Li[1], Pengping Xu[1,6], Tingting Zheng[2*], Chuan Xia[2,5,7], Jie Zeng[1,8]

[1]Hefei National Research Center for Physical Sciences at the Microscale, Key Laboratory of Strongly-Coupled Quantum Matter Physics of Chinese Academy of Sciences, Key Laboratory of Surface and Interface Chemistry and Energy Catalysis of Anhui Higher Education Institutes, Department of Chemical Physics, University of Science and Technology of China, 230026, Hefei, Anhui, P. R. China.

[2]School of Materials and Energy, University of Electronic Science and Technology of China, 611731, Chengdu, P. R. China.

[3]Institute of Fundamental and Frontier Sciences, University of Electronic Science and Technology of China, 611731, Chengdu, P. R. China.

[4]Key Laboratory of Soil Environment and Pollution Remediation, Institute of Soil Science, Chinese Academy of Sciences, 210008, Nanjing, P. R. China.

[5]Yangtze Delta Region Institute (Huzhou), University of Electronic Science and Technology of China, 313001, Huzhou, Zhejiang, P. R. China.

[6]Institute of Advanced Technology, University of Science and Technology of China, 230031, Hefei, Anhui, P. R. China.

[7]Research Center for Carbon-Neutral Environmental & Energy Technology, University of Electronic Science and Technology of China, 611731, Chengdu, P. R. China.

[8]School of Chemistry & Chemical Engineering, Anhui University of Technology, 243002, Ma'anshan, Anhui, P. R. China.

*Corresponding author: E-mail: ttzheng@uestc.edu.cn (T.Z.); chuan.xia@uestc.edu.cn (C.X.); zengj@ustc.edu.cn (J.Z.); †These authors contributed equally to this work.

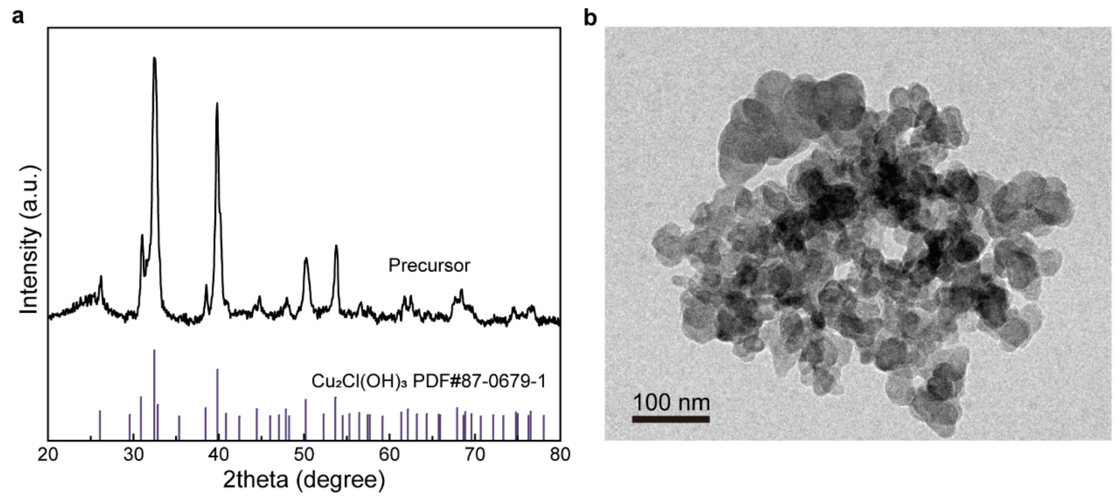

Supplementary Fig. 1 | XRD and TEM characterizations of the $Cu_2Cl(OH)_3$ precursor. (**a**) XRD pattern of the precursor, consistent with the standard $Cu_2Cl(OH)_3$. (**b**) TEM image of as-prepared precursor loaded on the activated carbon particles.

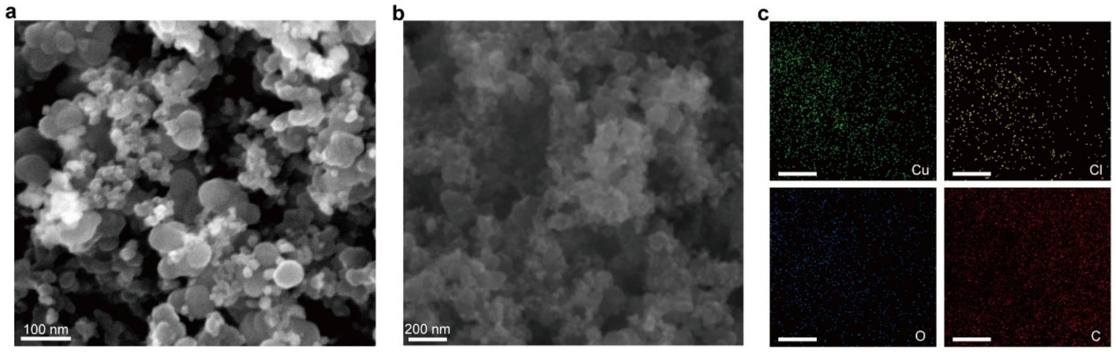

Supplementary Fig. 2 | SEM images of the $Cu_2Cl(OH)_3$ precursor. (**a**), (**b**) SEM images of the as-prepared precursor. Scale bar, (**a**) 100nm, (**b**) 200nm. (**c**) Corresponding EDS mapping of (**b**). The images showed that the $Cu_2Cl(OH)_3$ particles loaded on the activated carbon particles uniformly.

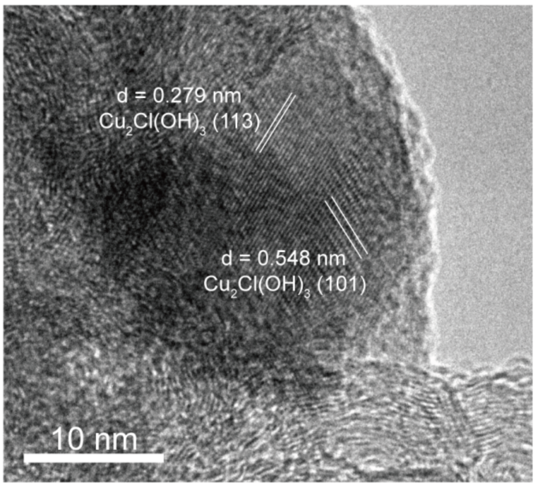

Supplementary Fig. 3 | HRTEM image of the $Cu_2Cl(OH)_3$ precursor. The lattice fringe spacing of 0.548 nm and 0.279 nm marked on the picture can be indexed to the (101) and (113) plane of $Cu_2Cl(OH)_3$, respectively.

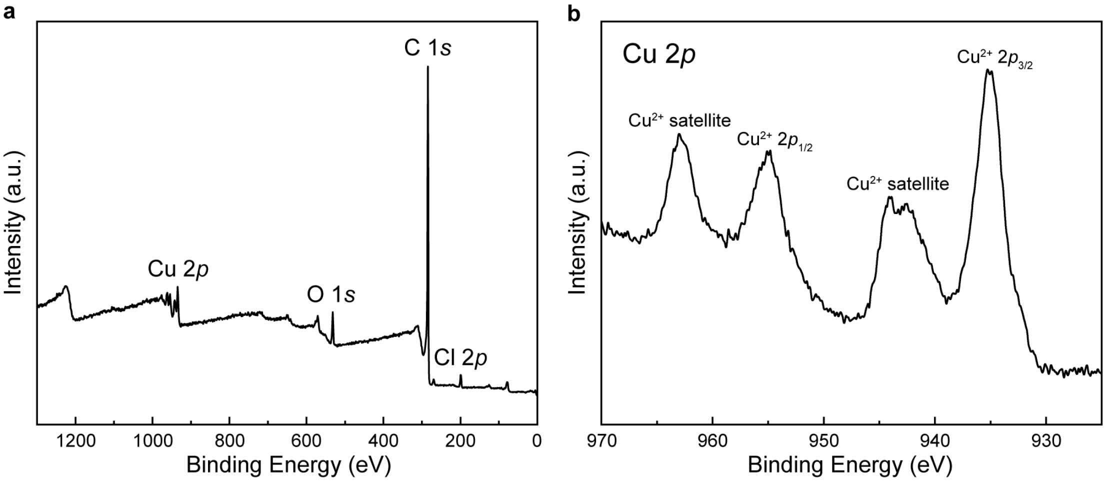

Supplementary Fig. 4 | XPS spectra of the $Cu_2Cl(OH)_3$ precursor. (**a**) Wide spectrum of the Cu precursor. (**b**) Cu 2*p* spectrum of the precursor, showing the valance state of Cu is +2.

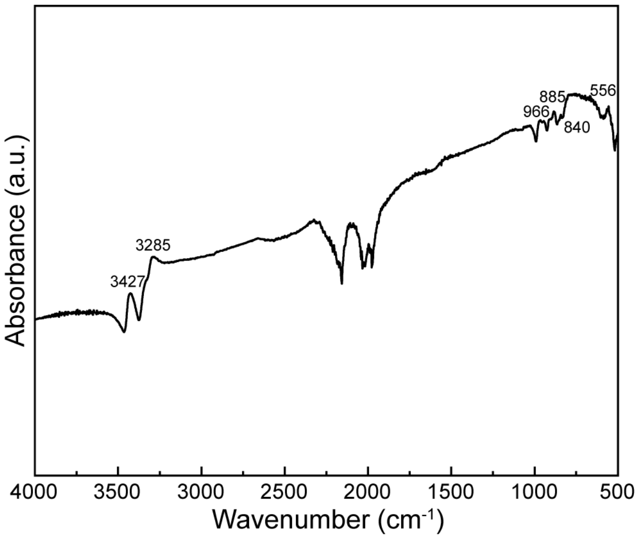

Supplementary Fig. 5 | Fourier transform infrared spectrum of the $Cu_2Cl(OH)_3$ precursor. Two absorption peaks at 3427 and 3285 cm^{-1} belonged to the hydroxyl stretching modes ν_1(O-H). The peaks at 840 to 966 cm^{-1} were related to δ_1(Cu-O-H) mode and the signal at 556 cm^{-1} was responding to the ν_1(O-Cu-O) mode. [*Catal. Sci. Technol.* **5**, 3970 (2015)]

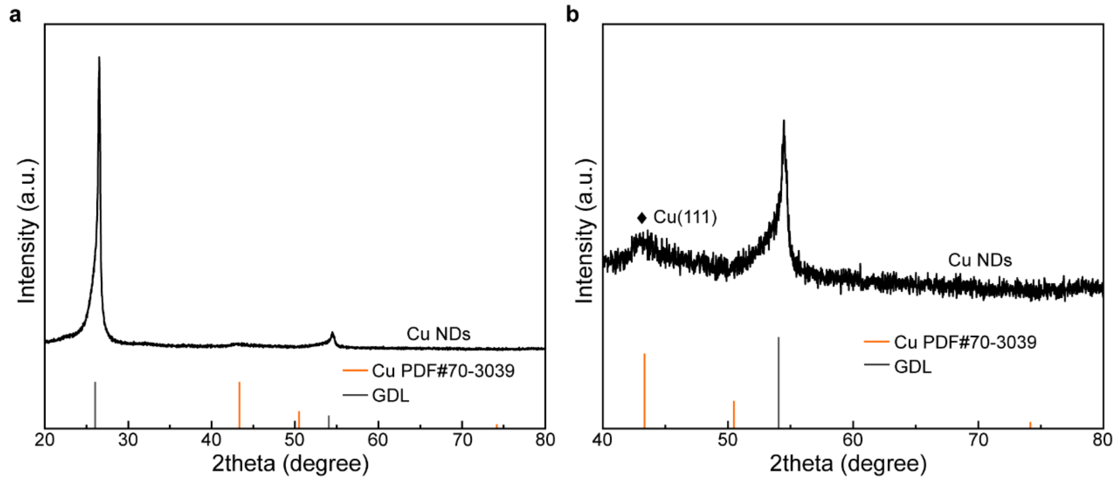

Supplementary Fig. 6 | XRD pattern of the Cu NDs on the GDL. (**a**) XRD pattern of Cu NDs with 2theta from 20° to 80°. (**b**) Enlarged region between 40° and 80°. The pattern suggested Cu NDs had the feature of metallic copper.

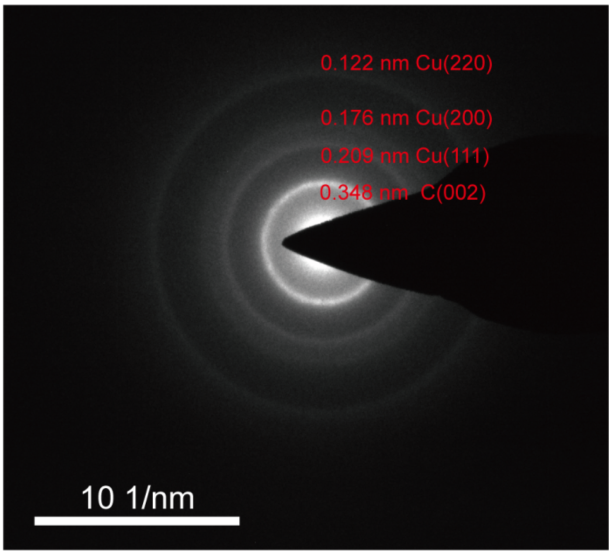

Supplementary Fig. 7 | Selected area electron diffraction pattern of Cu NDs. As specified in the diffraction pattern, the bright fringes were assigned to the (220), (200), (111) plane of copper, and (002) plane of activated carbon, respectively.

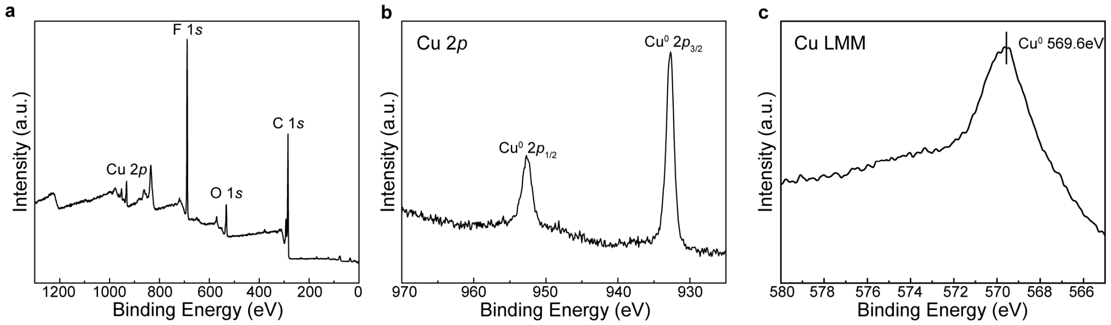

Supplementary Fig. 8 | XPS spectra of the Cu NDs on the GDL. (**a**) Wide spectrum, (**b**) Cu 2*p* spectrum and (**c**) Cu LMM auger spectrum of the Cu NDs. The spectra of Cu 2*p* and Cu LMM auger identified the state of Cu (0) in Cu NDs. The signal of F belonged to the PTFE in GDL.

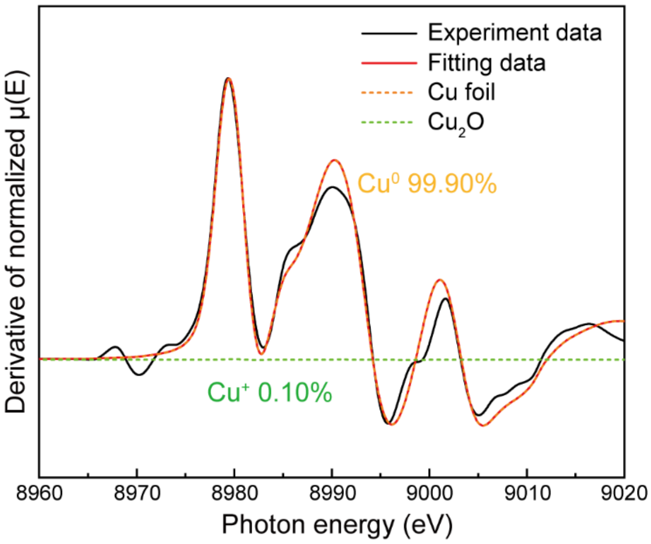

Supplementary Fig. 9 | Linear combination fitting for derivative of Cu *K*-edge XANES over Cu NDs at -0.7 V *vs.* RHE, corresponding to the data in Fig. 1e. Fitting range was between -20 eV to 40 eV, relative to E_0. Black and red solid lines denote experimental and fitting data, respectively. Fractions of Cu foil and Cu_2O making up the fitted spectra are shown by short dashed line. The percentage of Cu^0 in Cu NDs was 99.90% at the applied potential of -0.7 V *vs.* RHE (under maximum C_2H_4 FE), indicating the active sites of Cu NDs in electrocatalytic acetylene semihydrogenation were undercoordinated metallic Cu nanodots.

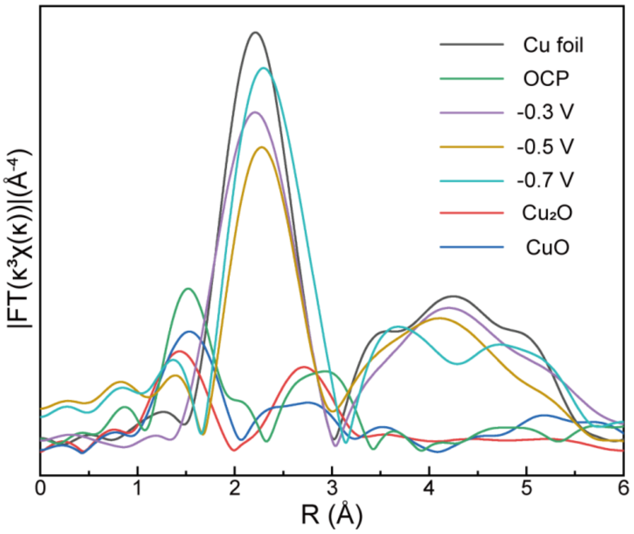

Supplementary Fig. 10 | *In situ* EXAFS at Cu *K*-edge in R space of Cu NDs, along with Cu foil, Cu_2O and CuO as references. The coordination environment of Cu NDs at working potentials was consistent with that of Cu foil, indicating that Cu NDs catalyst maintained a metallic feature under EASH conditions.

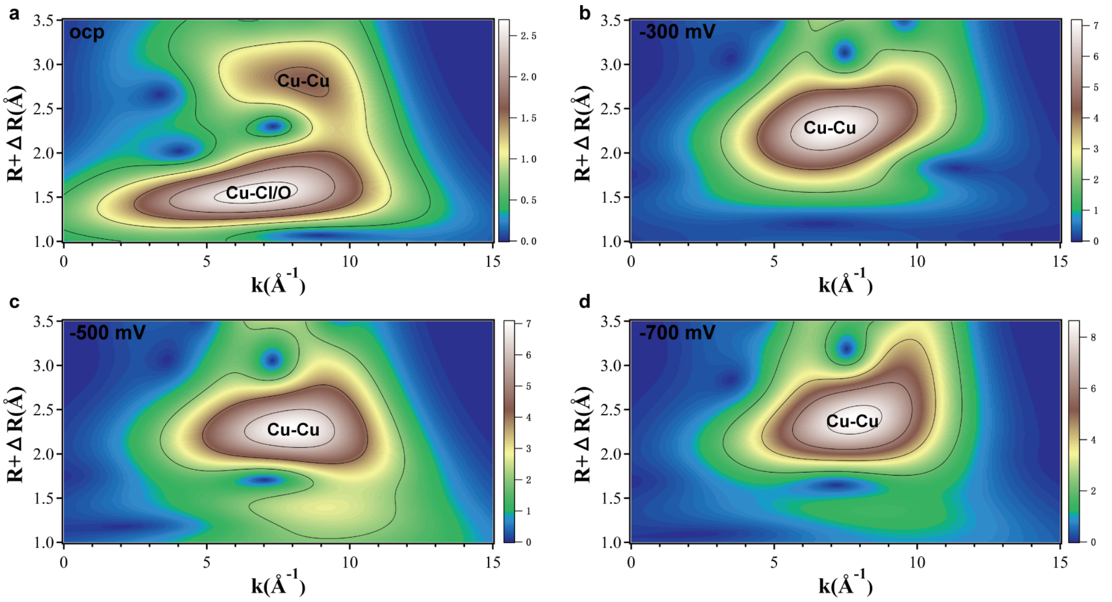

Supplementary Fig. 11 | *In situ* XAFS wavelet transforms (WT) for Cu NDs at different working potential *vs.* RHE, corresponding to the data in Supplementary Fig. 10. (**a**) OCP, open-circuit potential, (**b**) -300 mV, (**c**) -500 mV, (**d**) -700 mV. The *Y*-axis is the radical distance and the *X*-axis reflects the k-space resolution of the backscattering atom, from which we can clearly observe the bond length and coordination environment. It could be obviously observed that both Cu-Cu and Cu-Cl/O bond were present in the precursor while only Cu-Cu bond was found in Cu NDs.

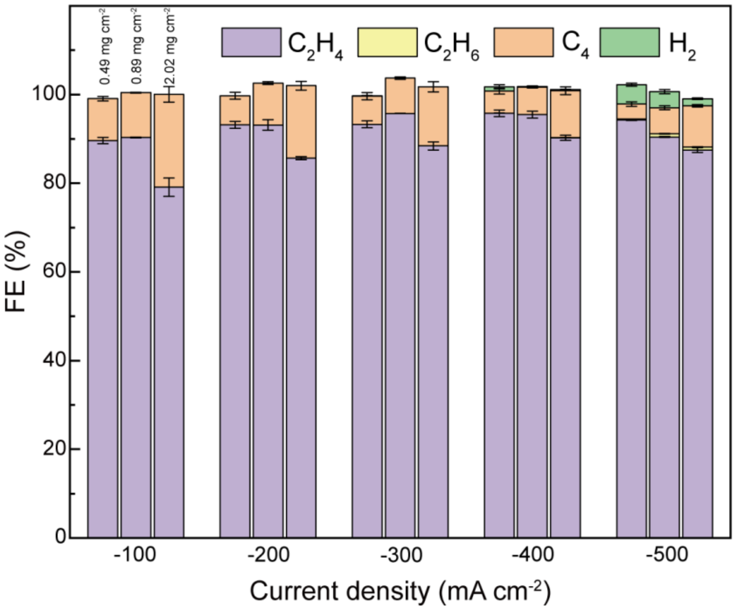

Supplementary Fig. 12 | FEs of electrocatalytic acetylene semihydrogenation products at different current densities over Cu NDs with different catalysts mass loading on gas diffusion electrode. Three bars from left to right correspond to the mass loading of 0.49, 0.89, and 2.02 mg cm^{-2} for catalyst precursors, respectively. The error bars correspond to the standard deviation of at least three independent measurements. The FE of coupling product C_4 would be improved with the increase of Cu NDs mass loading on GDE, ascribed to the impeded mass transfer which promotes the surface coverage of adsorbed acetylene and thus the coupling to C_4.

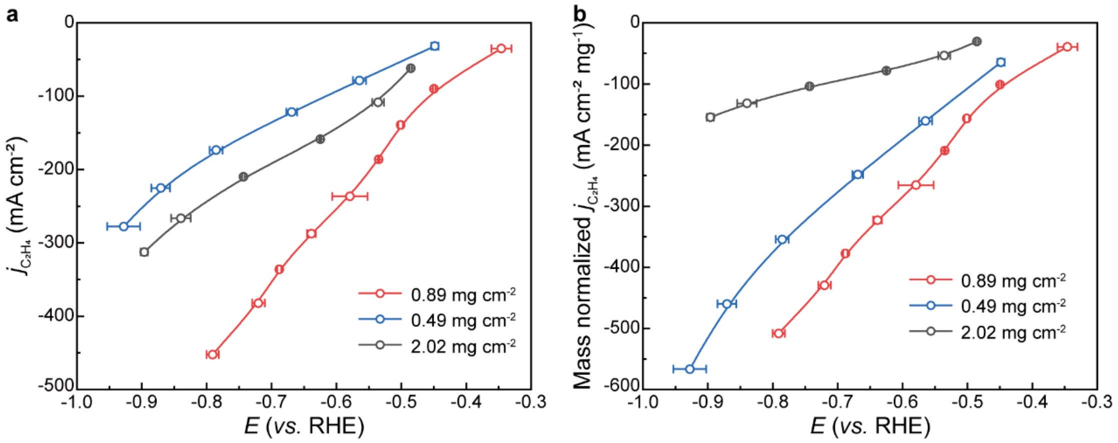

Supplementary Fig. 13 | Variation in the (a) ethylene partial current density and (b) mass normalized ethylene partial current density against applied potential over Cu NDs with different catalysts mass loading on gas diffusion electrode. The error bars correspond to the standard deviation of at least three independent measurements. Due to the fewer active sites, Cu NDs with the mass loading per geometric area of 0.49 mg cm^{-2} need a more negative potential to achieve the same ethylene partial current density as the Cu NDs with a mass loading of 0.89 mg cm^{-2} (Supplementary Fig. 13a). To rule out the mass loading effect and get a clear idea of the intrinsic activity, we further calculated the mass activity by normalizing the ethylene partial current density to the mass loading of Cu NDs. As shown in Supplementary Fig. 13b, apparently, Cu NDs with the mass loading of 0.89 mg cm^{-2} for precursor possessed the highest mass activity under the same potential. Thus, we controlled the mass loading of catalysts precursors on the gas diffusion electrode to be 1 mg cm^{-2}.

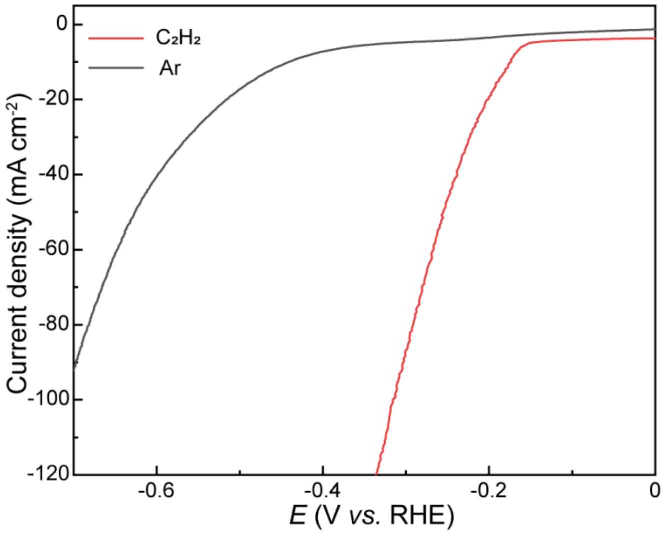

Supplementary Fig. 14 | Liner sweep voltammetry (LSV) of Cu NDs catalyst under different atmosphere. The polarized curves were collected in three-electrode flow-cell system using a scan rate of 20 mV s^{-1}. 4 mL min^{-1} 1 M KOH and 30 sccm C_2H_2 or Ar were provided. Cu NDs afforded a notably higher current density under pure acetylene than that under Ar, indicating the participation of C_2H_2 in the reaction.

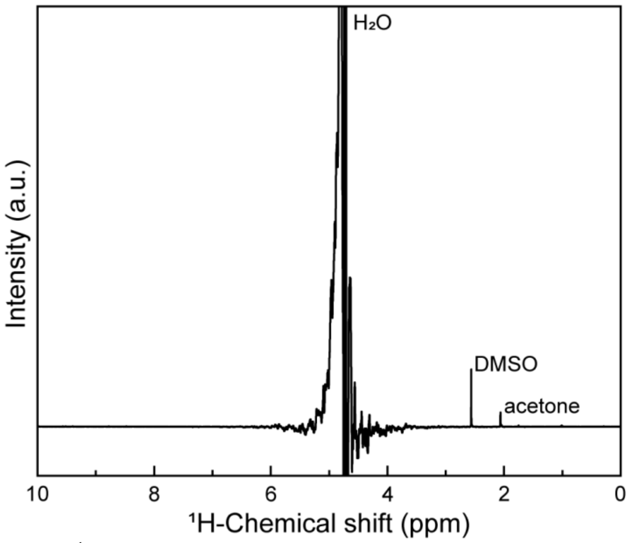

Supplementary Fig. 15 | ^{1}H NMR spectrum of EASH liquid products over Cu NDs. The liquid products of EASH were analyzed by NMR spectrum, in which none liquid product was detected. This spectrum was analyzed from the electrolyte at -300 mA cm^{-2}, and the acetone came from the cylinder of acetylene.

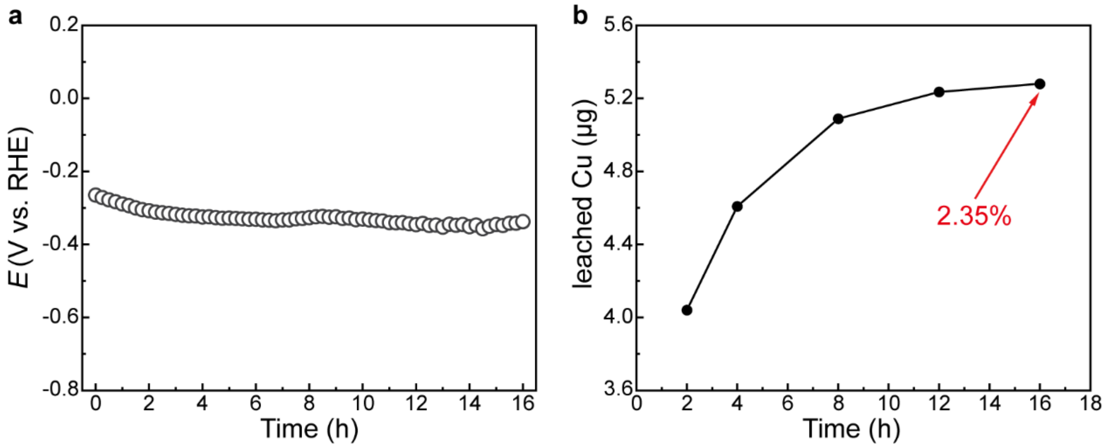

Supplementary Fig. 16 | Study on copper leaching under working condition in a flow cell. (**a**) Cathode potential against time at current density of -50 mA cm^{-2} over Cu NDs. (**b**) Mass of leached Cu at different time. Copper leaching of Cu NDs was evaluated in a typical flow cell system by inductively coupled plasma atomic emission spectroscopy (ICP-AES). 20 mL of 1 M KOH electrolyte was flowed through cathode and circulated by a peristaltic pump at a flow rate of 4 mL min^{-1}. In the process of constant current electrolysis at -50 mA cm^{-2}, 2 ml of electrolyte was taken out periodically and 1 M HCl was added for acidification. As depicted by Supplementary Fig. 16b, only 5.28 μg of Cu was leached (equating to a loss of 2.35% Cu at the working electrode) after 16 hours, suggesting that Cu NDs exhibited excellent stability under EASH electrolysis.

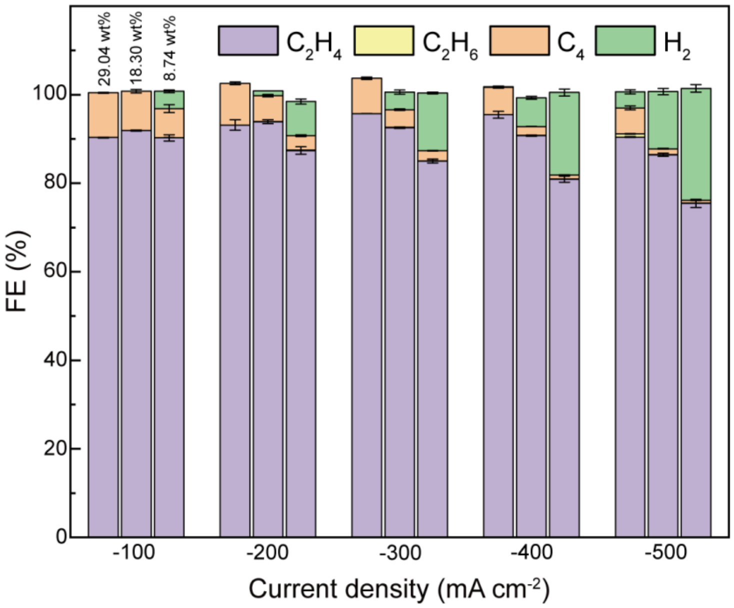

Supplementary Fig. 17 | FEs of electrocatalytic acetylene semihydrogenation products at different current densities over Cu NDs with different copper mass loading. Cu NDs catalysts with copper mass loading of 29.04 wt%, 18.30 wt%, and 8.74 wt% were synthesized by changing $CuCl_2$ feeding amount while maintaining the quantity of activated carbon the same. Three bars from left to right correspond to the copper mass loading of 29.04 wt%, 18.30 wt%, and 8.74 wt%, respectively. The error bars correspond to the standard deviation of at least three independent measurements. The selectivity of C_2H_4 would improve with the decrease of copper mass loading which might provide a higher degree of Cu sites with unsaturated coordination [*Nat. Commun.* **12**, 2932 (2021)]. However, decrease of the copper active sites density on the surface would weaken the acetylene semihydrogenation catalytic activity as well. Instead, more hydrogen evolution reaction happened over the catalysts with a lower copper mass loading.

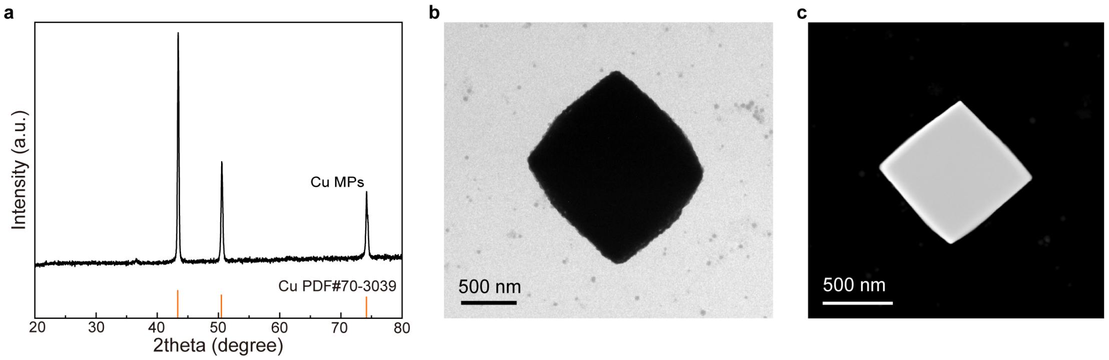

Supplementary Fig. 18 | Structural characterizations of Cu MPs. (**a**) XRD pattern of the Cu MPs, consistent with the standard Cu. (**b**) TEM image of Cu MPs. (**c**) HAADF image of Cu MPs. XRD pattern and electron microscope images showed that Cu MPs was the micron copper particles with a particle size of about 1 μm.

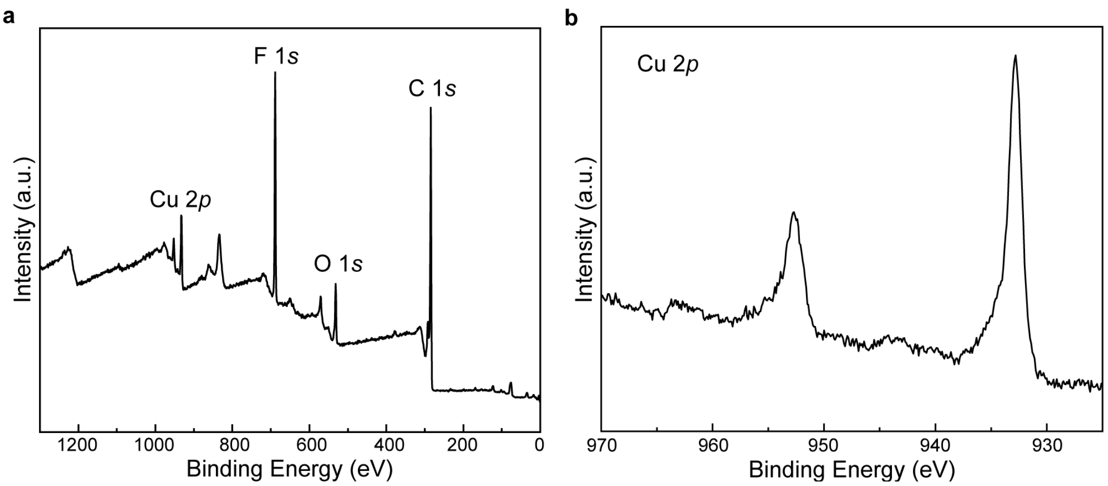

Supplementary Fig. 19 | XPS spectra of Cu MPs. (**a**) Wide spectrum and (**b**) Cu 2*p* spectrum of Cu MPs, showing the metallic state of Cu. The signal of F belonged to the PTFE in GDL.

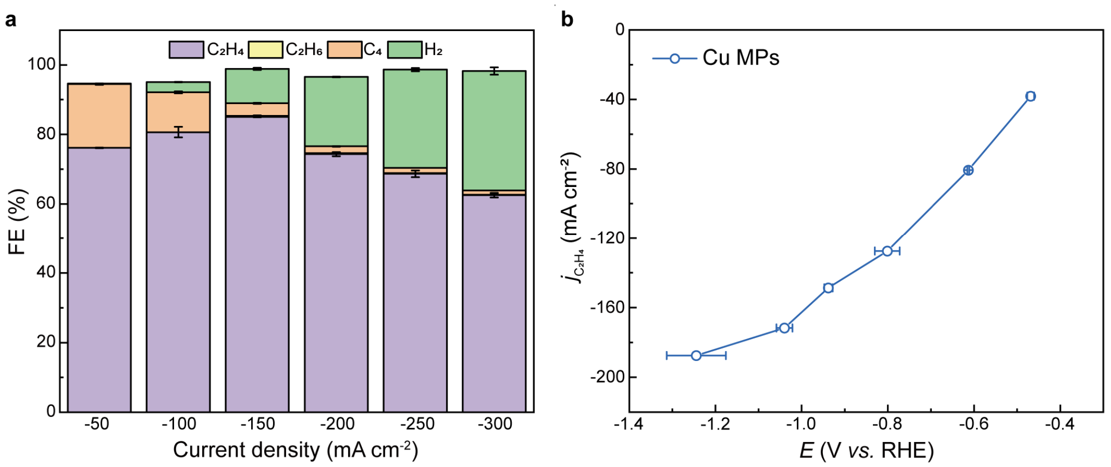

Supplementary Fig. 20 | Electrocatalytic acetylene semihydrogenation performance over Cu MPs under pure acetylene flow. (**a**) FEs of EASH products at different current densities and (**b**) the corresponding *j*–V curve of Cu MPs. The tests were conducted in 1 M KOH solution (4 ml min^{-1}) at room temperature under pure acetylene at a flow rate of 30 sccm.

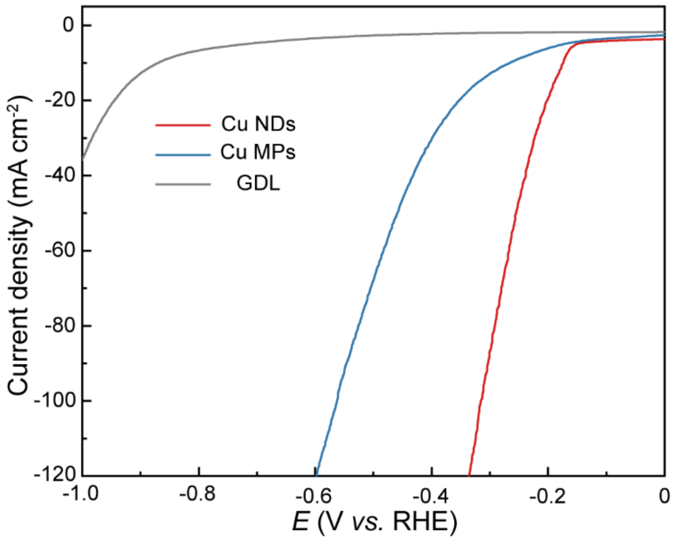

Supplementary Fig. 21 | Polarization curves of Cu NDs, Cu MPs and GDL under pure C_2H_2 flow. The LSV measurements were conducted in 1 M KOH solution (4 ml min^{-1}) under pure acetylene at the scan rate of 20 mV s^{-1}. Obviously, Cu NDs had a higher EASH intrinsic activity than Cu MPs and GDL.

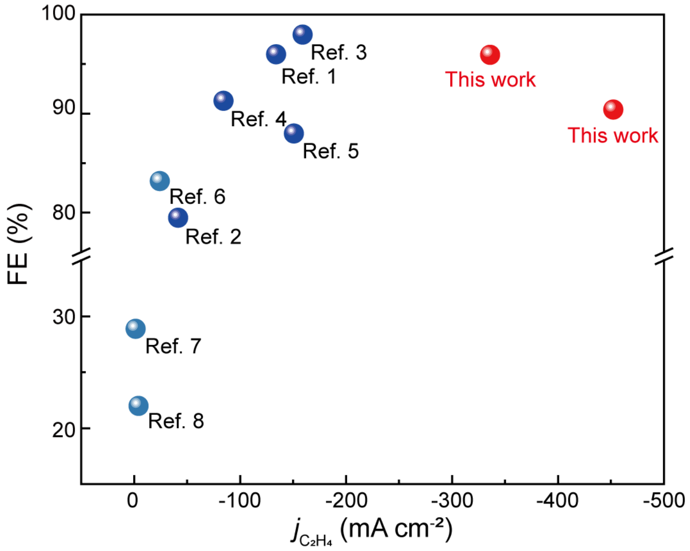

Supplementary Fig. 22 | Comparison in the FE_{C2H4} and ethylene partial current density over different reported catalysts. The translucent ball represents the catalyst performed in H-Cell. Cu NDs catalyst shows superiorities with respect to high j_{C2H4} and high FE_{C2H4} compared with previously reported catalysts. [Ref. 1: *Nat. Catal.* **4**, 565-574 (2021); Ref. 2: *Nat. Catal.* **4**, 557-564 (2021); Ref. 3: *Nat. Commun.* **12**, 6574 (2021); Ref. 4: *J. Mater. Chem. A* **10**, 6122 (2022); Ref. 5: *Chem. Eng. J.* **431**, 134129 (2022); Ref. 6: *Nat. Commun.* **12**, 7072 (2021); Ref. 7: *Electrochem. Commun.* **34**, 90-93 (2013); Ref. 8: *J. Electrochem. Soc.* **118**, 236 (1971)]. Cu NDs achieved an impressive C_2H_4 partial current density exceed 450 mA cm^{-2} with C_2H_4 FE of 90.4%, outperforming the reported state-of-art C_2H_2-to-C_2H_4 electrocatalysts, which indicated the outstanding intrinsic activity and selectivity towards electrocatlalytic acetylene semihydrogenation over undercoordinated Cu sites.

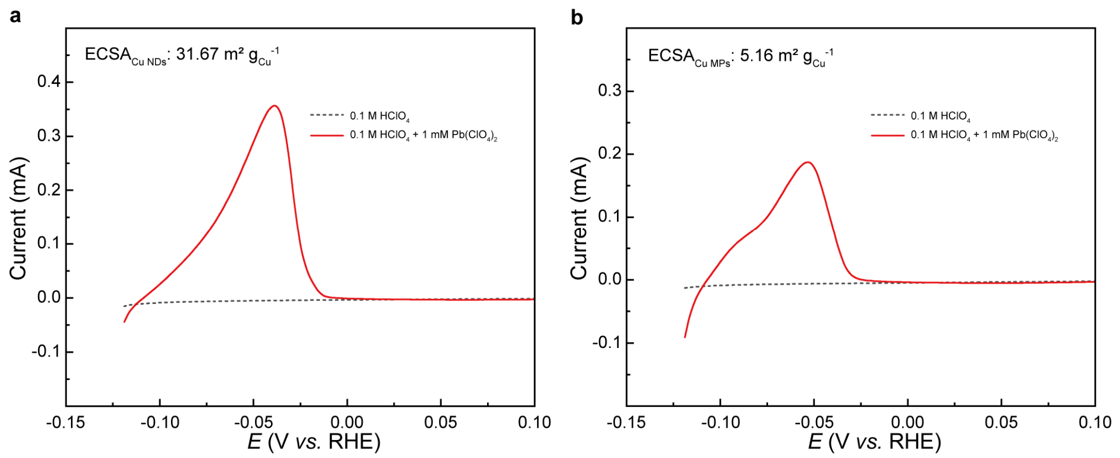

Supplementary Fig. 23 | Pb underpotential deposition (UPD) experiments for calculation of the electrochemical active surface area (ECSA) of Cu NDs and Cu MPs. Underpotential deposition of Pb was conducted to analyze ECSA in 0.1 M $HClO_4$ + 1 mM $Pb(ClO_4)_2$ solution, using data collected in 0.1 M $HClO_4$ as background. As shown in the figure, ECSAs were 31.67 $m^2\ g_{Cu}^{-1}$ and 5.16 $m^2\ g_{Cu}^{-1}$ for (**a**) Cu NDs and (**b**) Cu MPs, respectively.

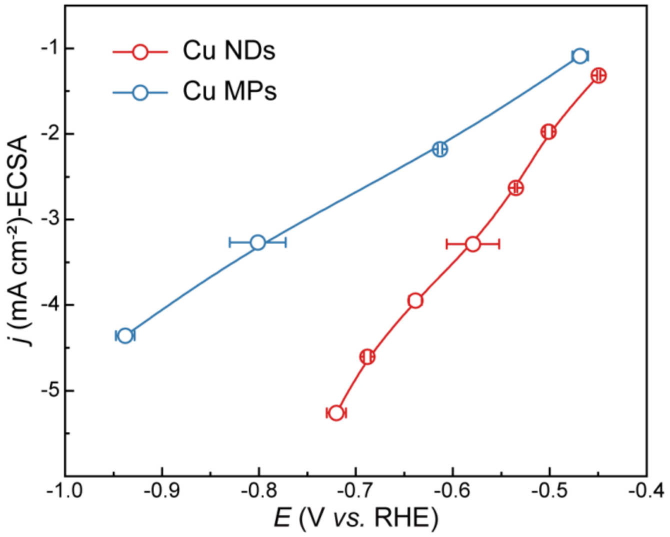

Supplementary Fig. 24 | ECSA-normalized current densities at different potential over Cu NDs and Cu MPs under pure C_2H_2 flow. The ECSA-normalized current densities of Cu NDs were clearly higher than those of Cu MPs at the same applied potentials, indicating the superior intrinsic catalytic activity of Cu NDs over Cu MPs.

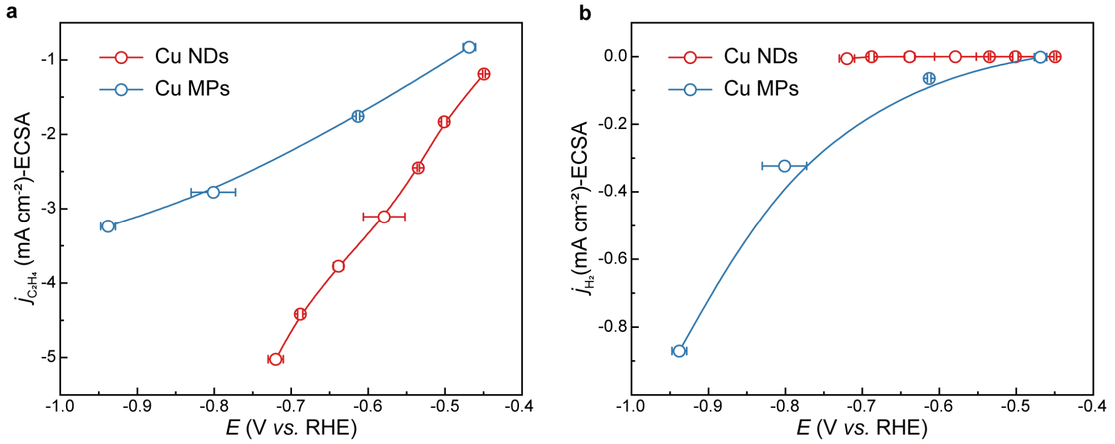

Supplementary Fig. 25 | ECSA normalized (a) C_2H_4 and (b) H_2 partial current densities at different potential over Cu NDs and Cu MPs under pure C_2H_2 flow, respectively. From these curves, we found that Cu NDs intrinsically favored selective C_2H_4 production, while Cu MPs was more preferable to HER.

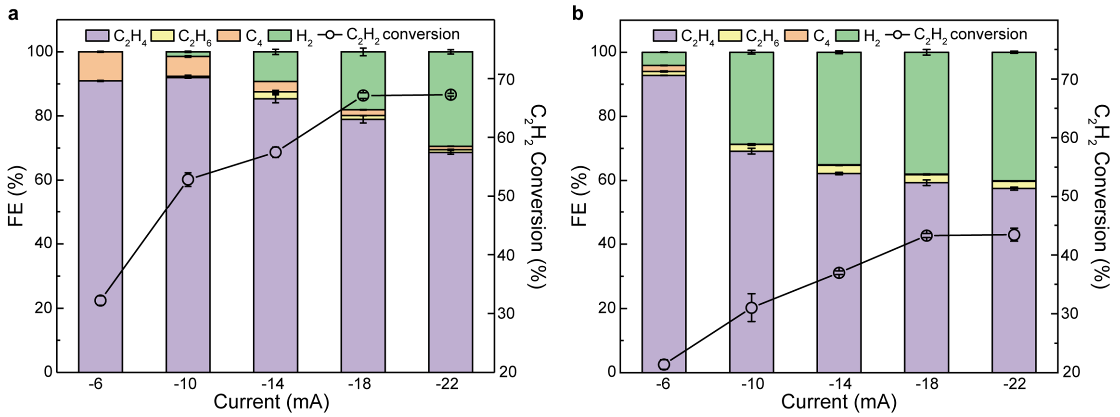

Supplementary Fig. 26 | FEs of EASH products at different currents and the corresponding C_2H_2 conversion over (a) Cu NDs and (b) Cu MPs. The experiments conducted using a three-electrode flow cell with a 0.75 cm^2 geometric electrode area at a flow rate of 20 sccm.

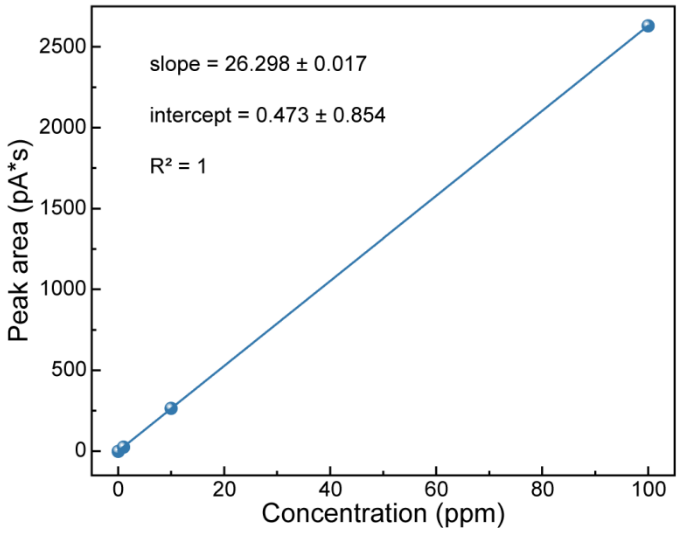

Supplementary Fig. 27 | Gas chromatograph (GC) calibration curve of C_2H_2. Confirming that the GC can detect the acetylene as concentration low as 1 ppm.

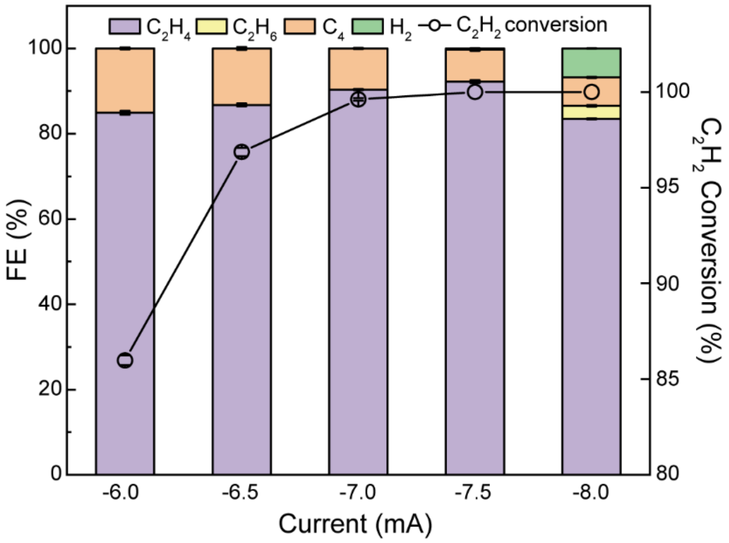

Supplementary Fig. 28 | FEs of EASH products at different currents and corresponding C_2H_2 conversion over Cu NDs. Using the two-electrode reactor (electrode area, 4 cm^2) under C_2H_4-riched simulated feed gas at a flow rate of 10 sccm.

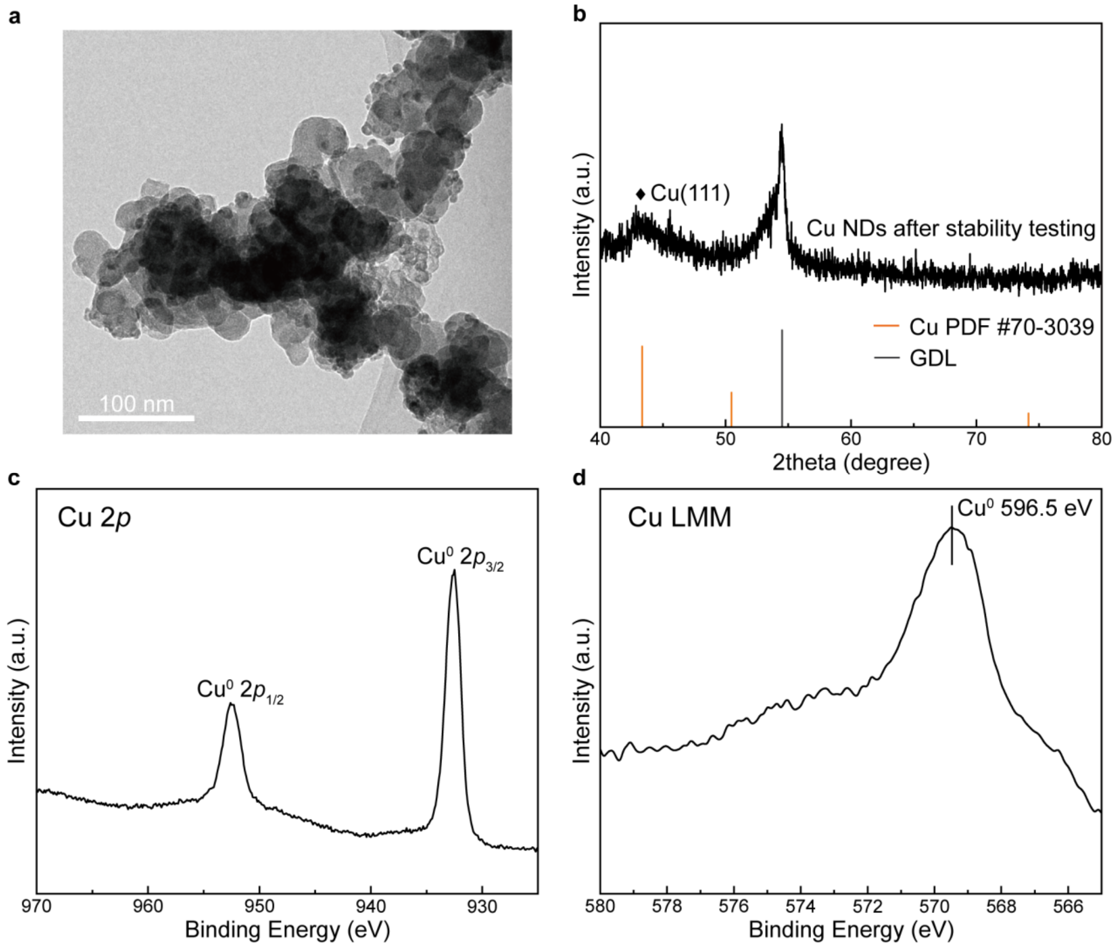

Supplementary Fig. 29 | Characterization of Cu NDs after the stability test. (**a**) TEM image. (**b**) XRD pattern of Cu NDs with 2theta from 40° to 80°. (**c**) Cu 2p XPS spectrum and (**d**) Cu LMM auger spectrum of Cu NDs. After the stability test in Fig. 4c, Cu NDs maintained its morphology, phase, and valence state, indicating the excellent material stability of Cu NDs.

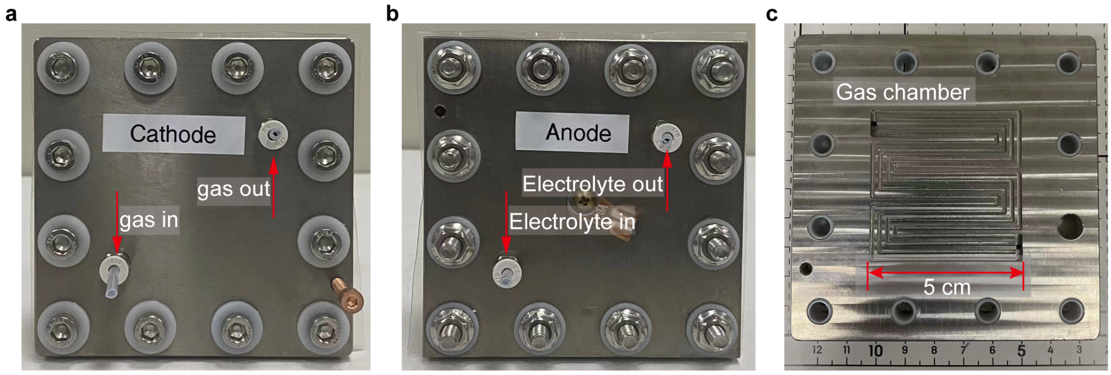

Supplementary Fig. 30 | Digital images of homemade MEA-type two-electrode reactor. (**a**) cathode, (**b**) anode, (**c**) gas chamber.

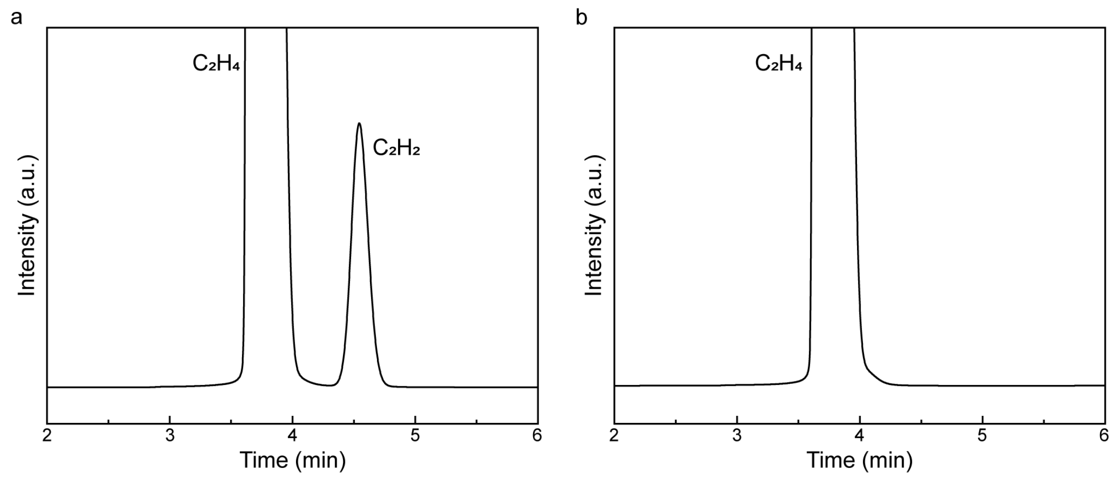

Supplementary Fig. 31 | GC curves of EASH over Cu NDs at the flow rate of 50 sccm in the homemade two-electrode reactor at the current of -50 mA. (**a**) Before reaction. (**b**) After reaction. Clearly, C_2H_2 impurities was removed completely after the reaction.

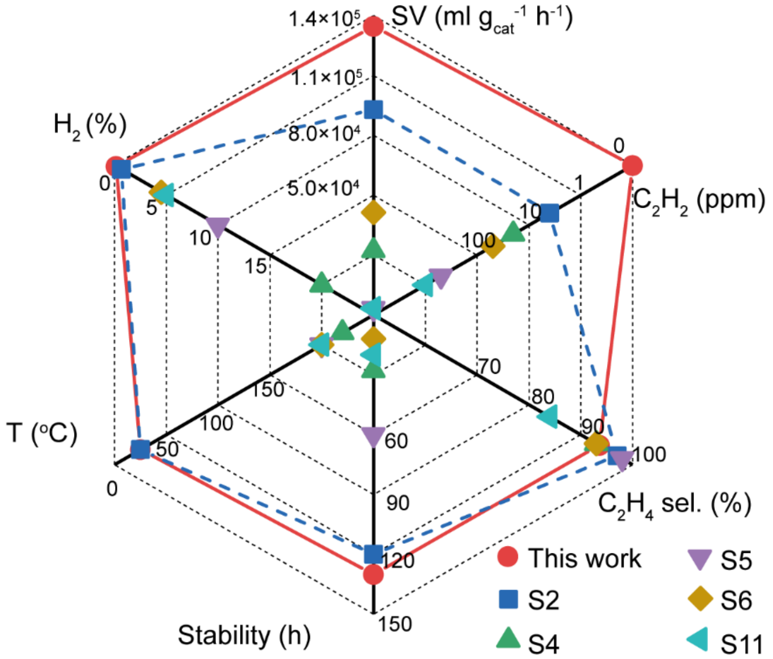

Supplementary Fig. 32 | Comparison of the acetylene semihydrogenation performance over Cu NDs with the previous reported state-of-the-art catalysts. SV, space velocity; sel., selectivity; T, temperature. All comparison data are from the references summarized in Supplementary Table 5. It clearly shows that Cu NDs exhibits distinct advantages over reported state-of-the-art catalysts in C_2H_2 removal.

Supplementary Table 1 | EXAFS fitting parameters at the Cu *K*-edge for Cu NDs and precursor (S_0^2 = 0.814).

Sample	Shell	N^a	R(Å)^b	σ^2(Å^2)^c	ΔE_0(eV)^d	R factor
Cu foil	Cu-Cu	12	2.54	0.0086	3.7	0.0006
Cu-OCP	Cu-O	3.4	1.96	0.0063	0.0	0.0004
	Cu-Cl	2.7	2.65	0.0065		
	Cu-Cu	2.2	3.15	0.0085		
	Cu-Cu	4.1	3.33	0.0085		
Cu-300mV	Cu-Cu	10.0	2.53	0.0077	3.7	0.0012
Cu-500mV	Cu-Cu	9.0	2.58	0.0092	8.5	0.0047
Cu-700mV	Cu-Cu	10.6	2.60	0.0085	2.6	0.0021

$^a N$: coordination numbers; $^b R$: bond distance; $^c\sigma^2$: Debye-Waller factors; $^d\Delta E_0$: the inner potential correction. *R* factor: goodness of fit. S_0^2 was set to 0.814 for Cu, according to the experimental EXAFS fit of Cu foil reference by fixing CN as the known crystallographic value.

Supplementary Table 2 | The original data of Fig. 2a. The data was analyzed based on three independent experiments.

Potential (V *vs.* RHE)	FE_{C2H4} (%)	FE_{H2} (%)	FE_{C4} (%)	FE_{C2H6} (%)	j (mA cm^{-2})	j_{C2H4}(mA cm^{-2})
-0.45	89.98	0.00	10.12	0.00	-100	-89.98
-0.50	92.85	0.00	7.60	0.00	-150	-139.28
-0.53	93.14	0.00	9.42	0.00	-200	-186.28
-0.58	94.58	0.00	4.72	0.00	-250	-236.45
-0.64	95.71	0.00	7.99	0.00	-300	-287.13
-0.69	95.94	0.00	7.00	0.00	-350	-335.79
-0.72	95.47	0.11	6.20	0.02	-400	-381.88
-0.74	90.89	1.55	7.34	1.04	-450	-409.01
-0.79	90.42	3.65	5.85	0.72	-500	-452.10

Supplementary Table 3 | Comparison of EASH performance under pure acetylene flow without ethylene.

Catalyst	FE (%)	$j_{C_2H_4}$ (mA cm^{-2})	Reactor	Reference
Cu NDs	95.94	-335.8	Flow Cell	This work
Cu NDs	90.42	-452.1	Flow Cell	This work
Cu dendrites	96.00	-134.0	Flow Cell	*Nat. Catal.* **4**, 565-574 (2021)
LD-Cu	79.50	-41.4	Flow Cell	*Nat. Catal.* **4**, 557-564 (2021)
NHC-Cu	98.00	-159.0	Flow Cell	*Nat. Commun.* **12**, 6574 (2021)
SA-Ni-NC	91.30	-84.2	Flow Cell	*J. Mater. Chem. A* **10**, 6122 (2022)
CoPc	88.00	-150.8	Flow Cell	*Chem. Eng. J.* **431**, 134129 (2022)
Cu MPs	83.20	-24.1	H-Cell	*Nat. Commun.* **12**, 7072(2021)
Cu	28.90	-4.6	H-cell	*Electrochem Commun.* **34**, 90-93 (2013)
Pt	22.00	-4.2	H-Cell	*J. Electrochem. Soc.* **118**, 236 (1971)

Supplementary Table 4 | The original data of Fig. 4d. The data was analyzed based on three independent experiments.

Flow rate	Selectivity of C_2H_4	Selectivity of C_4	H_2 volume	E_{cell}
10 sccm	86.58%	13.42%	0.01%	-1.67 V
20 sccm	90.03%	9.97%	0.08%	-1.76 V
30 sccm	91.82%	8.17%	0.10%	-1.80 V
40 sccm	93.08%	6.92%	0.13%	-1.85 V
50 sccm	93.59%	6.41%	0.16%	-1.89 V

Supplementary Table 5 | The comparison of acetylene semihydrogenation performance between electrocatalytic and thermocatalytic processes.

Catalyst	Gas composition	SV (ml g^{-1} h^{-1})	Residual C_2H_2 (ppm)	C_2H_4 Sel. （%）	Stability (h)	T (°C)	V_{H2} (%)	Ref.
Cu NDs	C_2H_2:C_2H_4:Ar= 0.5:20:79.5	1.35×10^5	0	94	130	25	0.18	This work
Cu dendrites	C_2H_2:C_2H_4 = 1:99	9.60×10^4	4	97	120	25	0.67	S1-*Nat. Catal.* **4**, 565-574 (2021)
LD-Cu	C_2H_2:C_2H_4:Ar= 0.5:20:79.5	/	5	90.1	4	25	0.08	S2-*Nat. Catal.* **4**, 557-564 (2021)
SA-Ni-NC	C_2H_2:C_2H_4 = 1:99	2.40×10^4	264	99	8	25	/	S3-*J. Mater. Chem. A* **10**, 6122 (2022)
NiCuFeGaGe /SiO_2	C_2H_2:C_2H_4:H_2:He = 1:10:10:39	3.00×10^4	20	93	28	220	16.67	S4-*Angew. Chem. Int. Ed.* **61**, 27 (2022)
Cu_1/ND@G	C_2H_2:C_2H_4:H_2:He = 1:10:10:69	3.00×10^3	500	98	60	200	10.00	S5-*Nat. Commun.* **10**, 4431 (2019)
$Al_{13}Fe_4$	C_2H_2:C_2H_4:H_2:He = 0.5:50:5:44.5	3.00×10^3	1000	84	20	200	5.00	S6-*Nat. Mater.* **11**, 690-693 (2012)
Pd-SAs	C_2H_2:C_2H_4:H_2:He = 0.5:50:5:44.5	1.20×10^3	200	93	/	120	5.00	S7-*Nat. Nanotechnol.* **13**, 856-861 (2018)
PdZn-1.2 @ZIF-8C	C_2H_2:C_2H_4:H_2:Ar = 0.65:50:5:44.35	4.80×10^4	2200	80	12	115	5.00	S8-*Adv. Mater.* **30**, 33 (2018)
$CuPd_{0.006}/SiO_2$	C_2H_2:C_2H_4:H_2:He = 1:20:20:59	6.00×10^4	/	85	24	160	20.00	S9-*ACS Catal.* **7**, 1491-1500 (2017)
Pd@SOD	C_2H_2:C_2H_4:H_2:N2 = 0.6:0:6:93.4	3.00×10^4	12	94	15	150	6.00	S10-*Angew. Chem. Int. Ed.* **58**, 23 (2019)
$Ni_3ZnC_{0.7}$/oCNT	C_2H_2:C_2H_4:H_2:He = 0.5:20:4.5:75	4.80×10^4	50	93	12	200	4.50	S11-*Nat. Commum.* **11**, 3324 (2020)
Pd-Pt/SiO_2	C_2H_2:H_2:He = 1.67:3.33:95	1.35×10^5	167	40	/	80	3.33	S12-*Science* **362**, 560-564 (2018)
$Co_2Mn_{0.5}Fe_{0.5}Ge$	C_2H_2:C_2H_4:H_2:He = 0.1:10:40:69	2.00×10^4	10	85	/	200	40.00	S13-*Sci. Adv.* eaat6063(2018)
Graphene	C_2H_2:C_2H_4:H_2:N_2 = 0.1:0.9:3:94	1.46×10^4	210	92	/	110	3.00	S14-*Nat. Commun.* **5**, 5291-5300 (2014)
Pd-Zn/ZnO	C_2H_2:C_2H_4:H_2:He = 2:40:20:38	1.8×10^5	1200	90	20	80	20.00	S15-*ACS Catal.* **6**, 1054-1061 (2016)
Ni@CeO_2	C_2H_2:C_2H_4:H_2:He = 1:20:10:69	6×10^4	/	90	30	180	10.00	S16-*J. Am. Chem. Soc.* **132**, 4321-4327(2010)

SV, space velocity; Sel., selectivity; T, temperature; V_{H2}, volume of H_2.

Supplementary Table 6 | Comparison of acetylene impurities removal performance between our work and reported electrocatalysts. Superior to state-of-art reported electrocatalysts, Cu NDs could convert C_2H_2 impurities completely at a relatively high space velocity of 1.35×10^5 ml g^{-1} h^{-1}. In addition, Cu NDs was capable to operate over 130 h with a C_2H_4 selectivity of 94%. In conclusion, Cu NDs not only possesses excellent intrinsic C_2H_2-to-C_2H_4 catalytic activity and selectivity, but also has ability to completely remove the acetylene impurities efficiently.

Catalyst	Space velocity (ml g^{-1} h^{-1})	Residual C_2H_2 (ppm)	C_2H_4 selectivity (%)	Stability (h)	Reference
Cu NDs	1.35×10^5	0	94	130	This work
Cu dendrites	9.60×10^4	4	97	120	*Nat. Catal.* **4**, 565-574 (2021)
LD-Cu	/	5	90.1	4	*Nat. Catal.* **4**, 557-564 (2021)
NHC-Cu	9.6×10^5	30	99	100	*Nat. Commun.* **12**, 6574 (2021)
SA-Ni-NC	2.4×10^4	264	99	8	*J. Mater. Chem. A* **10**, 6122 (2022)

Supplementary Note 1

Techno-economic analysis

In order to envision the industrial prospect of Cu NDs, we did a quantitatively techno-economic analysis of our strategy for crude ethylene purification according to previous method [*Ind. Eng. Chem. Res.* **57**, 2165-2177 (2018)], assuming a C_2H_4 purification rate of 100,000 kg per day. According to Fig. 4e, electrode area of 25 cm^2 was required to remove acetylene impurities at a flow rate of 50 ml min^{-1} and the current was 50 mA with a cell voltage of -1.89 V.

The total flow rate needed is:

$$total\ flow\ rate = 100{,}000\frac{kg}{day} * \frac{day}{1{,}440min} * \frac{mol}{28g} * \frac{1{,}000g}{kg} * \frac{24{,}500ml}{mol} = 60{,}763{,}889\frac{ml}{min} \quad (S1)$$

The total electrode area needed is given by the required flow rate:

$$total\ electrode\ area = 60{,}763{,}889\frac{mL}{min} * \frac{min}{50ml} * 25cm^2 * \frac{m^2}{10{,}000cm^2} = 3{,}038.2m^2 \quad (S2)$$

The total current needed for our system is:

$$total\ current = \frac{3{,}038.2m^2}{25cm^2} * \frac{10{,}000cm^2}{m^2} * 50mA * \frac{A}{1{,}000mA} = 6{,}0764A \quad (S3)$$

The power needed is given from *P=UI*:

$$power = 1.89V * 60{,}764A * \frac{kW}{1{,}000W} = 114.8kW \quad (S4)$$

For the full cell reaction of $2C_2H_2 + 2H_2O \rightarrow 2C_2H_4 + O_2$ or $2H_2O \rightarrow 2H_2 + O_2$ the water consumption rate is:

$$water\ consumption\ rate = 60{,}764A * \frac{2}{4e^- * 96{,}485\frac{C}{mol}} * \frac{0.018kg}{mol} * \frac{0.2642gal}{kg} * \frac{86{,}400s}{day} = \frac{129.4gal}{day} \quad (S5)$$

Capital costs

From the DOE (U. S. Department of Energy) H_2A analysis (the hydrogen analysis project of DOE) for central grid electrolysis, the electrolyzer cost for the stack component is 250.25 USD per kilowatt. The reference electrolyzer is operated at 0.175 A cm^{-2} and 1.75 V. The installation factor is 1.2. Thus, the cost per area for the reference electrolyzer is:

$$Ref.electrolyzer\ cost = \frac{\$250.25}{kW} * \frac{0.175A}{cm^2} * 1.75V * \frac{10^4 cm^2}{m^2} * \frac{kW}{1{,}000W} * 1.2$$
$$= \frac{\$919.7}{m^2} \quad \text{(S6)}$$

Thus, the electrolyzer capital cost is given by multiplying the total area:

$$Electrolyzer\ capital\ cost = 3{,}038.2m^2 * \frac{\$919.7}{m^2} = \$2{,}794{,}232.5 \quad \text{(S7)}$$

From the H_2A, the balance of plant (BoP) capital cost is 35% of the total cost, while the stack is 65%:

$$BoP\ capital\ cost = \$2{,}794{,}232.5 * \frac{0.35}{0.65} = \$1{,}504{,}586.7 \quad \text{(S8)}$$

Because of the hydrogen byproduct, the PSA (Pressure Swing Adsorption) capital cost is calculated by scaling the reference cost to the total flow rate:

$$PSA\ capital\ cost = \$1{,}989{,}043 * \left(\frac{60{,}763{,}889\frac{ml}{min} * \frac{m^3}{1{,}000{,}000ml}}{1{,}000\frac{m^3}{hr} * \frac{hr}{60min}}\right)^{0.7}$$
$$= \$4{,}919{,}284.5 \quad \text{(S9)}$$

So, the sum of capital costs is $9,218,103.7.

Operating costs

The electricity cost is calculated from the power and the price of electricity, which is supposed to be 0.03 USD per kilowatt-hour. Thus, the electricity cost for 1 year (350 days) is:

$$Electricity\ cost = 114.8kW * \frac{\$0.03}{kWh} * \frac{24hr}{day} * \frac{350day}{year} = \frac{\$28{,}929.6}{year} \quad \text{(S10)}$$

The cost of the PSA is calculated by scaling the reference cost to the flow rate:

$$PSA\ operating\ cost = \frac{0.25kWh}{m^3} * 3{,}645.8\frac{m^3}{hr} * \frac{8{,}400hr}{year} * \frac{\$0.03}{kWh} = \frac{\$229{,}685.4}{year} \quad \text{(S11)}$$

The maintenance cost is assumed 2.5% of capital cost per year (from H2A):

$$Maintenance\ cost = (\$2{,}794{,}232.5 + \$1{,}504{,}586.7 + \$4{,}919{,}284.5) * \frac{0.025}{year}$$
$$= \frac{\$230{,}452.6}{year} \quad \text{(S12)}$$

The cost of the water for 1 year is:

$$water\ cost = \frac{129.4gal}{day} * \frac{\$0.0054}{gal} * \frac{350day}{year} = \frac{\$244.6}{year} \quad \text{(S13)}$$

The cost of the catalysts is included in the electrolyzer cost, thus, the total cost of C_2H_4 purification in the first 5 years is:

$Total\ cost$

$$= Capital\ cost + Operating\ cost$$
$$= Capital\ cost + (electricity + PSA + maintenance + water) * year$$
$$= \$9{,}218{,}103.7 + (\$28{,}929.6 + \$229{,}685.4 + \$230{,}452.6 + \$244.6) * 5$$
$$= \$11{,}664{,}664.7 \quad \text{(S14)}$$

Take above together, in the case of the C_2H_4 purification rate of 100,000 kg per day, the total cost in the first 5 years is 11,664,664.7 USD, corresponding to 66.7 USD per ton. As an indispensable and essential part of ethylene production, the cost of ethylene purification in our system is just about 5.4% of ethylene price ($1,230), indicating our system with Cu NDs as catalyst has the potential to industrialize.
